# RNA sequencing identifies global transcriptional changes in peripheral CD4^+^ cells during active oesophagitis and following epicutaneous immunotherapy in eosinophilic oesophagitis

**DOI:** 10.1002/cti2.1314

**Published:** 2021-07-22

**Authors:** Melanie A Ruffner, Zhe Zhang, Kelly Maurer, Amanda B Muir, Antonella Cianferoni, Kathleen E Sullivan, Jonathan M Spergel

**Affiliations:** ^1^ Division of Allergy and Immunology The Children's Hospital of Philadelphia Philadelphia PA USA; ^2^ Department of Pediatrics The Perelman School of Medicine at University of Pennsylvania Philadelphia PA USA; ^3^ Department of Biomedical and Health Informatics The Children's Hospital of Philadelphia Philadelphia PA USA; ^4^ Division of Gastroenterology, Hepatology, and Nutrition The Children's Hospital of Philadelphia Philadelphia PA USA

**Keywords:** CD4‐positive lymphocytes, eosinophilic oesophagitis, food allergy, immunotherapy, interferon response, RNA sequencing

## Abstract

**Objective:**

There are no disease‐modifying therapies for the treatment of eosinophilic oesophagitis (EoE), which is driven by non‐IgE‐mediated allergic inflammation. A recent clinical trial of milk epicutaneous immunotherapy (EPIT) has shown initial promise, with 47% of treated EoE patients tolerating milk without recurrence of disease. Mechanisms of EPIT in EoE have not been studied in humans. Here, we identify transcriptional changes in the peripheral CD4^+^ T‐cell compartment during active EoE and following EPIT.

**Methods:**

RNA isolation, sequencing and integrative data analysis were performed on peripheral CD4^+^ T cells isolated from 15 of 20 patients enrolled in a clinical trial of EPIT for EoE. Gene expression changes in peripheral CD4^+^ T cells were examined during diet therapy and following trial of milk antigen EPIT.

**Results:**

We identify 244 differentially expressed genes in peripheral blood CD4^+^ cells of EoE patients consuming versus those eliminating milk, and 129 DEGs in CD4^+^ cells were isolated after EPIT versus after placebo (FDR ≤ 0.05). Gene set enrichment analysis identifies enrichment of hallmark interferon‐α and interferon‐γ response pathways in peripheral CD4^+^ T cells from EoE patients during active disease on a milk‐containing diet. We demonstrate overlap of this gene signature with the altered gene expression signature seen in EoE patient biopsy tissue. EPIT therapy response is associated with significant enrichment in pathways related to T‐cell receptor signalling (*P* = 1.16 × 10^−14^), antigen presentation and costimulation, and cytokine signalling (*P* = 1.11 × 10^−16^), as well as upregulation of genes associated with regulatory T‐cell function.

**Conclusions:**

EoE is associated with distinct global transcriptional changes in CD4^+^ T cells, one feature of which is an IFN response signature. Clinically favorable response to EPIT is likely multifactorial but is associated with a distinct transcriptional profile in peripheral CD4^+^ cells supporting the hypothesis that EPIT alters peripheral CD4^+^ responses in EoE patients.

## Graphical abstract

There are no approved disease‐modifying treatments for eosinophilic esophagitis (EoE), but recent clinical trials of milk antigen epicutaneous immunotherapy allowed 47% of patients to tolerate milk without oesophageal inflammation. We investigated peripheral CD4^+^ T‐cell gene expression changes in patients during diet therapy and after the trial of epicutaneous immunotherapy using RNA sequencing. EoE is associated with distinct global transcriptional changes in CD4^+^ T cells, one feature of which is an IFN response signature. Clinically favorable response to EPIT is likely multifactorial but is associated with a distinct transcriptional profile in peripheral CD4^+^ cells, supporting the hypothesis that EPIT alters peripheral CD4^+^ responses in EoE.

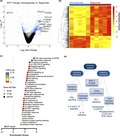

## Introduction

Eosinophilic oesophagitis (EoE) is an inflammatory disorder of the oesophagus occurring in approximately 1 out of every 2000 people in the United States.^1^, ^2^ EoE is a major cause of GI morbidity and significant direct healthcare expenditures.^3^ In contrast to immunoglobulin E (IgE)‐mediated food allergies, disease resolution in EoE is rare and most patients require lifelong treatment.^4^ The existing treatment options for EoE are limited and do not alter the underlying immunopathology. Therefore, when treatments are discontinued, inflammation recurs.

EoE is a multifactorial inflammatory disease of the oesophageal mucosa that is triggered by food allergens. EoE is thought to result from a combination of inherited risks and environmental exposures.^5^, ^6^, ^7^, ^8^ Over 90% of EoE patients will improve on a strict elemental diet, suggesting that antigen‐specific adaptive immunity plays a central role in EoE pathogenisis.^9^ Several lines of data implicate CD4^+^ helper T cells in the pathogenesis of EoE. Murine studies demonstrate that CD3^+^ T cells are critical for the establishment of experimental EoE.^10^, ^11^ Additionally, EoE patients have evidence of Th2 immune activation in the mucosa.^12^ Th2 cytokines interleukin (IL)‐4, IL‐5 and IL‐13 are expressed at high levels in EoE biopsy tissue, and single‐cell RNA sequencing has demonstrated elevated numbers of CD3^+^ Th2‐like GATA3^+^ cells in the oesophageal mucosa.^13^, ^14^ Further, peripheral CD4^+^CD154^+^ cells from EoE patients upregulate Th2 cytokine production of Th2 cytokines IL‐5 and IL‐13 when exposed to food antigens during *in vitro* antigen stimulation assays.^15^, ^16^ These data implicate aberrant antigen‐specific T‐cell responses as a key contributor to the pathogenesis of EoE.

Food allergen immunotherapy has shown considerable promise for the treatment of IgE‐mediated food allergy. Although EoE is largely a food allergen‐driven disease, the pathogenesis of EoE is non‐IgE‐mediated and therefore food allergy immunotherapy in EoE has not been utilised. Spergel *et al*.^17^ have recently reported that epicutaneous delivery of milk antigen reduced oesophageal eosinophilia in paediatric EoE patients upon milk reintroduction, suggesting that epicutaneous immunotherapy (EPIT) may hold promise as a modality for food allergen immunotherapy in EoE. EPIT for milk may have broad applicability as cow's milk is the most frequent causative food antigen in EoE, and studies of EPIT in IgE‐mediated food allergy patients thus far suggest an overall favorable safety profile.^18^, ^19^, ^20^ Preclinical studies of EPIT in EoE suggest the CD4^+^ T‐cell compartment is targeted by this therapy. Data from animal models demonstrate that EPIT induces epigenetic changes in milk antigen‐specific CD4^+^ T cells, shifting the overall T helper cell balance away from a T helper 2 (Th2) phenotype.^21^, ^22^ Further, EPIT therapy induced regulatory T cells with unique trafficking properties which would permit access to the GI tract in addition to the skin.^23^ In combination, it is hypothesised that these changes underlie the reduced mucosal inflammation seen with antigen rechallenge in these models. However, this has not been previously investigated in EoE patients, and it is unknown whether similar changes would occur in humans.

Here, we investigate transcriptional changes in the peripheral CD4^+^ T cells of paediatric EoE patients undergoing milk desensitisation with EPIT. Patients enrolled in the phase IIa study of milk EPIT for EoE were separately enrolled into this optional research investigation in order to test the hypothesis that alterations in human peripheral CD4^+^ T‐cell responses underlie the mechanism of epicutaneous desensitisation in EoE patients.^17^ RNA sequencing (RNA‐seq) was used to compare transcriptome‐wide changes in CD4^+^ gene expression in EoE patients at three time points: during active disease, during remission while excluding milk from the diet and again at the conclusion of the trial following EPIT therapy. Our analyses focused on several questions: (1) What are the transcriptional changes in the peripheral CD4^+^ T‐cell population during active inflammation in EoE compared to an inactive state achieved by dietary therapy? (2) What are the transcriptional changes in the peripheral CD4^+^ T‐cell population that are associated with EPIT therapy? And (3) how do these changes relate to the tissue pathophysiology of EoE?

## Results

### Patient cohort and response to EPIT

**Figure 1:**
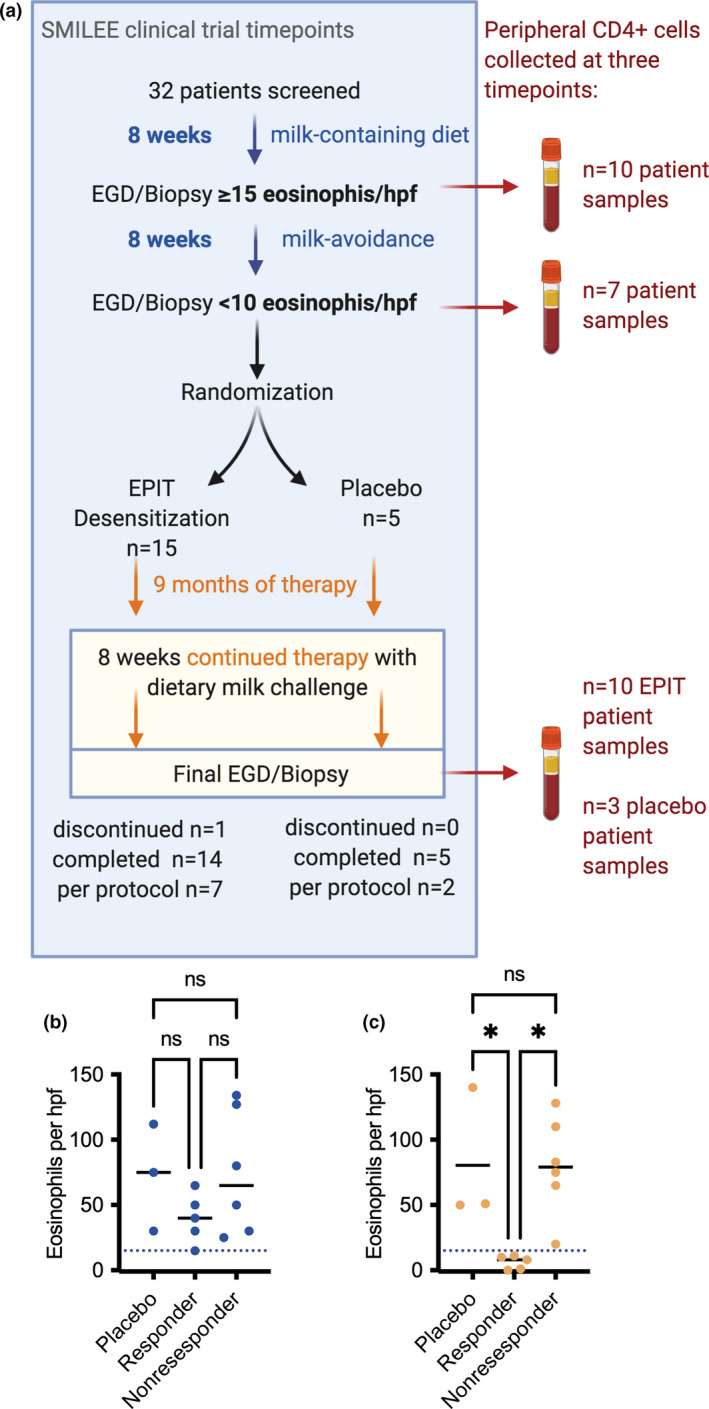
Overview of Study of Efficacy and Safety of Viaskin Milk for milk‐induced EoE (SMILEE) trial time points and patient endoscopy results relevant to this study. **(a)** SMILEE time points and enrolment numbers. The numbers of peripheral CD4^+^ T‐cell samples collected are shown at each of three visit time points: during the screening phase while on a milk‐containing diet (*n* = 10 patient samples), during the screening phase while avoiding milk (*n* = 7 patient samples) and following 11 months of EPIT therapy (*n* = 3 placebo and *n* = 10 EPIT patient samples). **(b)** Results from the ‘on‐milk’ pretreatment screening biopsy (eosinophils per high‐powered field show no difference in biopsy results between patients randomised to placebo or active therapy). **(c)** Post‐treatment biopsy results for the subset of placebo (*n* = 3) and active EPIT therapy patients in this research study. EPIT responder group had post‐EPIT biopsy < 15 eos hpf^−1^ (*n* = 5) and nonresponder (*n* = 6) had post‐EPIT biopsy ≥ 15 eos hpf^−1^. Blue dotted line = 15 eos hpf^−1^ cut‐off; black lines represent group mean. ANOVA F = 7.78 *P* = 0.0078, * denotes *P* ≤ 0.05, Tukey HSD test.

Fifteen out of 20 SMILEE study patients provided longitudinal blood samples during this research study. These patients had a median age of 11 years and were 68% male, and 86% had at least one comorbid atopic condition (Table 1, demographics). Patients underwent screening endoscopy to ensure milk‐elicited oesophageal inflammation (Figure 1a). The patients enrolled in our research study did not have significantly different levels of mucosal inflammation prior to study randomisation that could explain differences in therapeutic response (Figure 1b). We collected a total of 11 peripheral blood samples from patients randomised to active EPIT treatment and three samples from patient randomised to placebo. The mean post‐treatment placebo group biopsy was 80.3 ± 51.7 eos hpf^−1^. Within the subset of patients participating in this study, five patients randomised to EPIT had a favorable clinical response following 11 months of EPIT, with resolution of mucosal inflammation during the milk challenge (Figure 1c, classified as responder group; mean eos hpf^−1^ 6.0 ± 5.1). In contrast, six patients continued to have active mucosal inflammation at this final milk challenge despite receiving active EPIT therapy (Figure 1c, classified as nonresponder group, mean ± SD, 80.2 ± 37.6).

### CD4^+^ gene expression signature in EoE

We set out to test the hypothesis that CD4^+^ T‐cell responses are critical in the mechanism of EPIT in EoE patients. Peripheral CD4^+^ T cells were isolated from paediatric EoE patients at the three study time points (Figure 1a), and total RNA was isolated for sequencing.

**Figure 2:**
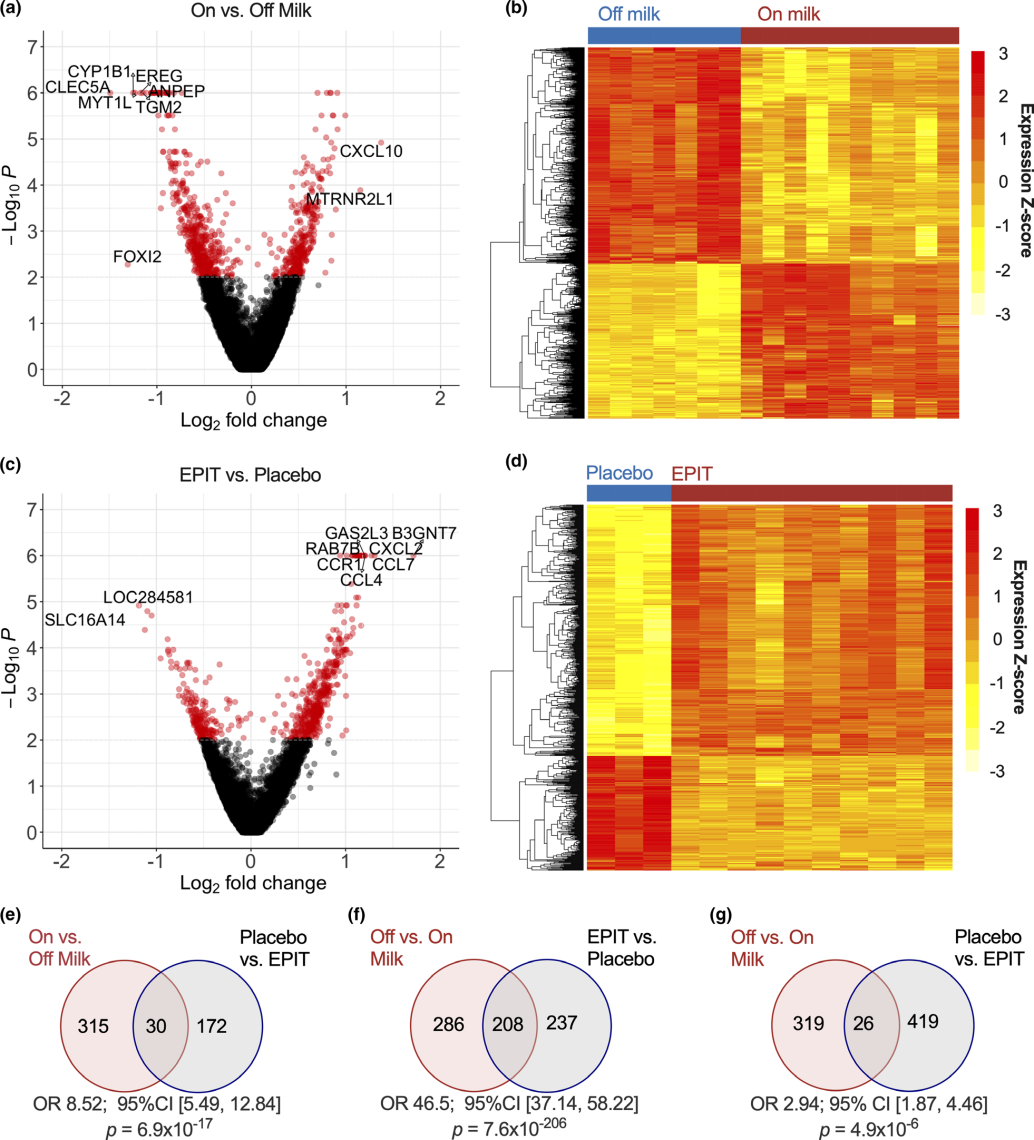
Differential gene expression in peripheral CD4^+^ cells identified by RNA sequencing. Differentially expressed genes (DEGs) from the peripheral CD4^+^ T cells of EoE patients collected while consuming versus abstaining from a cow's milk‐containing diet (‘on‐ > off‐milk’, *n* = 10 on‐milk and *n* = 7 off‐milk patient samples) represented in **(a)** volcano plot with *P*‐value (Log_10_) versus fold change expression (Log_2_). Red data points denote *P* ≤ 0.01. **(b)** Heatmap of DEGs at from on‐ versus off‐milk comparison at *P* ≤ 0.01. The legend displays mapping to row‐wise *Z*‐scores. **(c)** Volcano plot of DEGs from peripheral CD4^+^ T cells of EoE patients in the active EPIT versus placebo treatment groups (‘EPIT > placebo’, *n* = 3 placebo and *n* = 10 EPIT patient samples). Red data points denote *P* ≤ 0.01. **(d)** Heatmap of DEGs from active EPIT versus placebo treatment comparison at *P* ≤ 0.01. The legend displays mapping to row‐wise *Z*‐scores. **(e)** Venn diagrams of shared DEGs at *P* ≤ 0.01 between on‐ > off‐milk and placebo > EPIT, **(f)** off‐ > on‐milk and EPIT > placebo, **(g)** off‐ > on‐milk and placebo > EPIT with significance of gene overlapping determined using Fisher's exact test. The off‐ < on‐milk and placebo > EPIT comparison is nonsignificant (*P* = 0.3).

We first compared the gene expression of CD4^+^ T cells from patients consuming cow's milk versus those on milk‐avoidance diet to assess peripheral CD4^+^ gene expression changes associated with active inflammation on cow's milk‐containing diet in EoE patients. Within this set of samples, six sets of paired patient samples were analysed in addition to five additional patient samples from whom paired sample collection was not feasible (Supplementary table 1). A total of 839 transcripts were differentially expressed at a threshold cut‐off of *P* ≤ 0.01 (Figure 2a), with 345 genes upregulated and 494 downregulated in the on‐ compared to off‐milk comparison. The majority of these had modest fold change difference, with 36.9% of transcripts over 1.5‐fold change difference between groups. As shown in Figure 2b, we observe a relatively consistent gene expression pattern in CD4^+^ cells of EoE patients on milk‐containing diet and when not consuming milk. Similarly, we observe 647 differentially expressed transcripts (*P* ≤ 0.01 cut‐off) in CD4^+^ from patients on EPIT therapy compared to those who were on placebo (Figure 2c). Of these, 445 were upregulated in EPIT‐treated patients (EPIT > placebo) and 202 were downregulated in EPIT‐treated patients (placebo > EPIT), with increased interpatient heterogeneity in gene expression (Figure 2d).

We examined the degree of expression overlap between the EPIT vs. placebo and on‐ vs. off‐milk comparisons, hypothesising that patients in the placebo group have CD4^+^ gene expression signatures comparable to those seen from the on‐milk, pre‐EPIT EoE patient group . In these comparisons, there is significant overlap of DEGs in the on‐milk > off‐milk and placebo > EPIT comparison (odds ratio 8.52; 95% CI [5.49, 12.84] *P* = 6.9e‐17, Figure 2e). Further, we observe that the converse comparison of off‐ > on‐milk genes to EPIT > placebo is also highly significant (OR 46.5; 95% CI [37.14, 58.22], *P* = 7.6e‐206, Figure 2f). We also examined alternative comparisons, but there is no significant overlap between the off‐milk < on‐milk and placebo > EPIT comparison (*P* = 0.3008), and the off > on and placebo > EPIT DEG comparison has 26 overlapping genes with a lower odds ratio (OR = 2.94; 95% CI = [1.87, 4.46], *P* = 4.9e‐06, Figure 2g). In total, this suggests there is a set of common differentially expressed genes (DEGs) in the peripheral CD4^+^ T‐cell compartment that characterises both the uninflamed and inflamed state in EoE patients. In our dataset, this is composed of 208 DEGs.

We selected the cut‐off of *P* ≤ 0.01 for differential gene expression analysis because the primary goal of our analyses was to identify potential pathways or mechanisms of interest in the peripheral CD4^+^ T‐cell response in EoE and to EPIT in particular. Alternatively, if using the Benjamini–Hochberg method with an FDR cut‐off of 0.05, we identify 244 DEGs altered during milk‐associated active EoE disease. Of these, 97 transcripts were upregulated and 147 were downregulated at the time of the active milk diet endoscopy (Supplementary table 2). Using the same FDR cut‐off of 0.05, we observe a total of 129 DEGs, with 106 upregulated and 23 downregulated transcripts in the comparison of CD4^+^ cells from EPIT versus placebo treated patients (Supplementary table 3).

### Gene set enrichment analysis points to alterations in CD4^+^ immune function following EPIT

**Figure 3:**
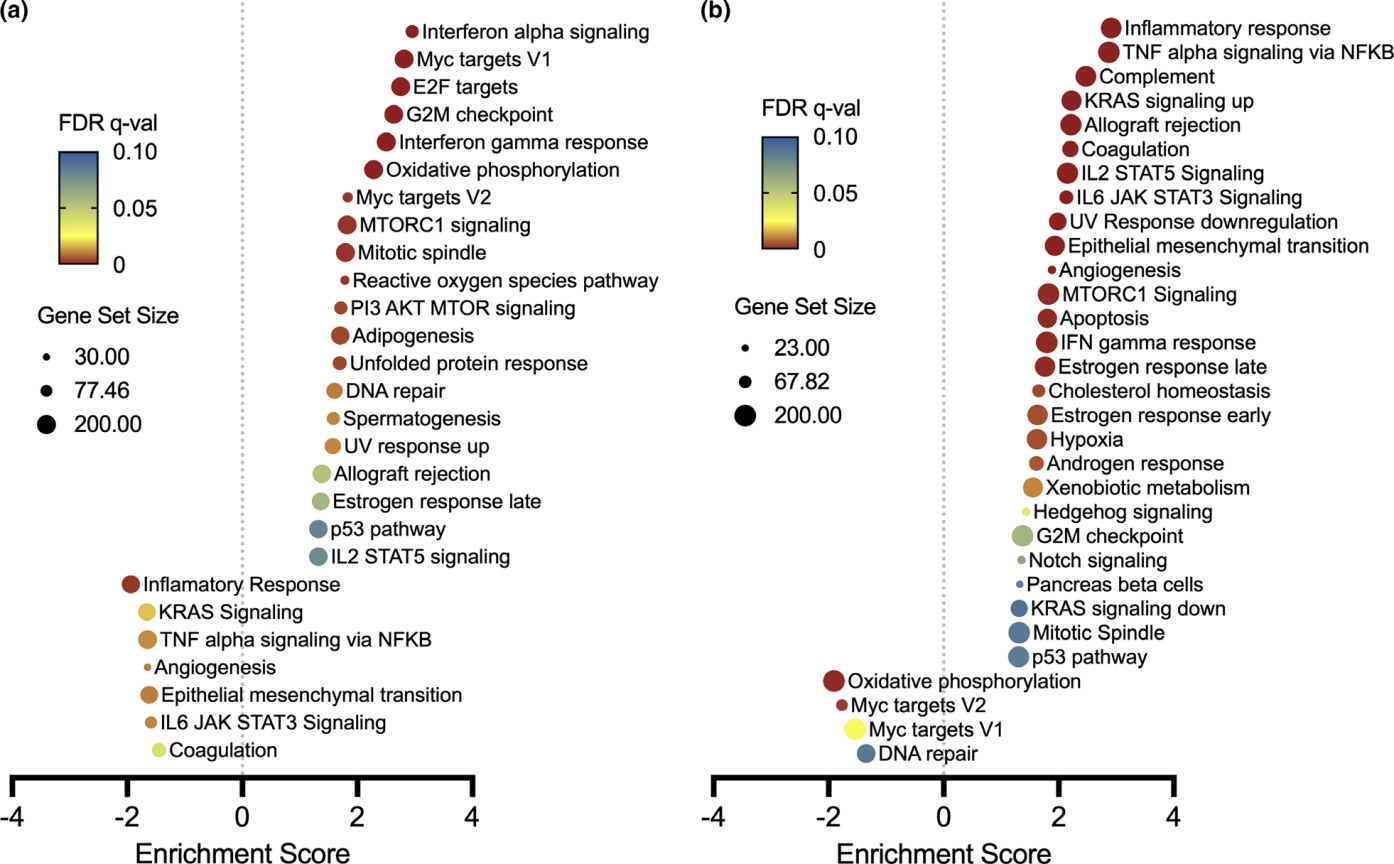
Functional enrichment analysis identifies potential peripheral CD4^+^ pathways associated with EPIT therapy. Hallmark GSEA from RNA‐Seq experiments performed on CD4^+^ from EoE patients **(a)** consuming versus abstaining from a cow's milk‐containing diet (on‐ > off‐milk) and **(b)** in the active EPIT versus placebo treatment groups (EPIT > placebo). Pathway results with FDR *q*‐val < 0.1 are plotted by normalised enrichment score (NES). Dot size represents the number of genes in the pathway, and colour represents the GSEA FDR as represented in the legend.

We next performed gene set enrichment analysis (GSEA) to examine differences between these conditions as differentially expressed pathways using the MSigDB Hallmark gene set collection. In EoE patients before and after milk elimination diet (on‐ > off‐milk), we observe enrichment of interferon (IFN)‐α and IFN‐γ response pathways (Figure 3a, Supplementary figure 1). We have previously reported that paediatric and adult EoE patients have a conserved IFN signature in biopsy tissue,^24^ but in this study, we did not observe an IFN signature in whole blood samples from EoE patients compared to non‐EoE controls. However, here, our transcriptional studies demonstrate that IFN pathway genes are enriched in the isolated CD4^+^ T cells of EoE patients.

We next performed GSEA in the EPIT > placebo sample comparison (Figure 3b). Because all patients were consuming milk at the time of the final study biopsy, we expect that pathway enrichment observed in the EPIT versus placebo comparison would relate to the effects of therapy. IFN‐γ response is enriched; however, we do not see a significant enrichment in IFN‐α response pathways. In the comparison of the patients on EPIT versus placebo, we observe positive enrichment in the ‘TNFα signalling via NF‐κB’ pathway and ‘Epithelial Mesenchymal Transition’ pathways (Figure 3b, Supplementary figure 2). These are driven by modest upregulation in the expression of several key cytokines including *INHBA, TGFBR3, IL6, CXCL1, CXCL2 and CCL4*. As a feature in these pathways, we observe modest upregulation in *FOSB* (fold change 1.68, FDR = 0.042), suggesting that altered expression of the AP‐1 transcription factor in peripheral T cells may be a potential mechanism of action of EPIT therapy in EoE patients.

### Gene signature associated with favorable EPIT response

**Figure 4:**
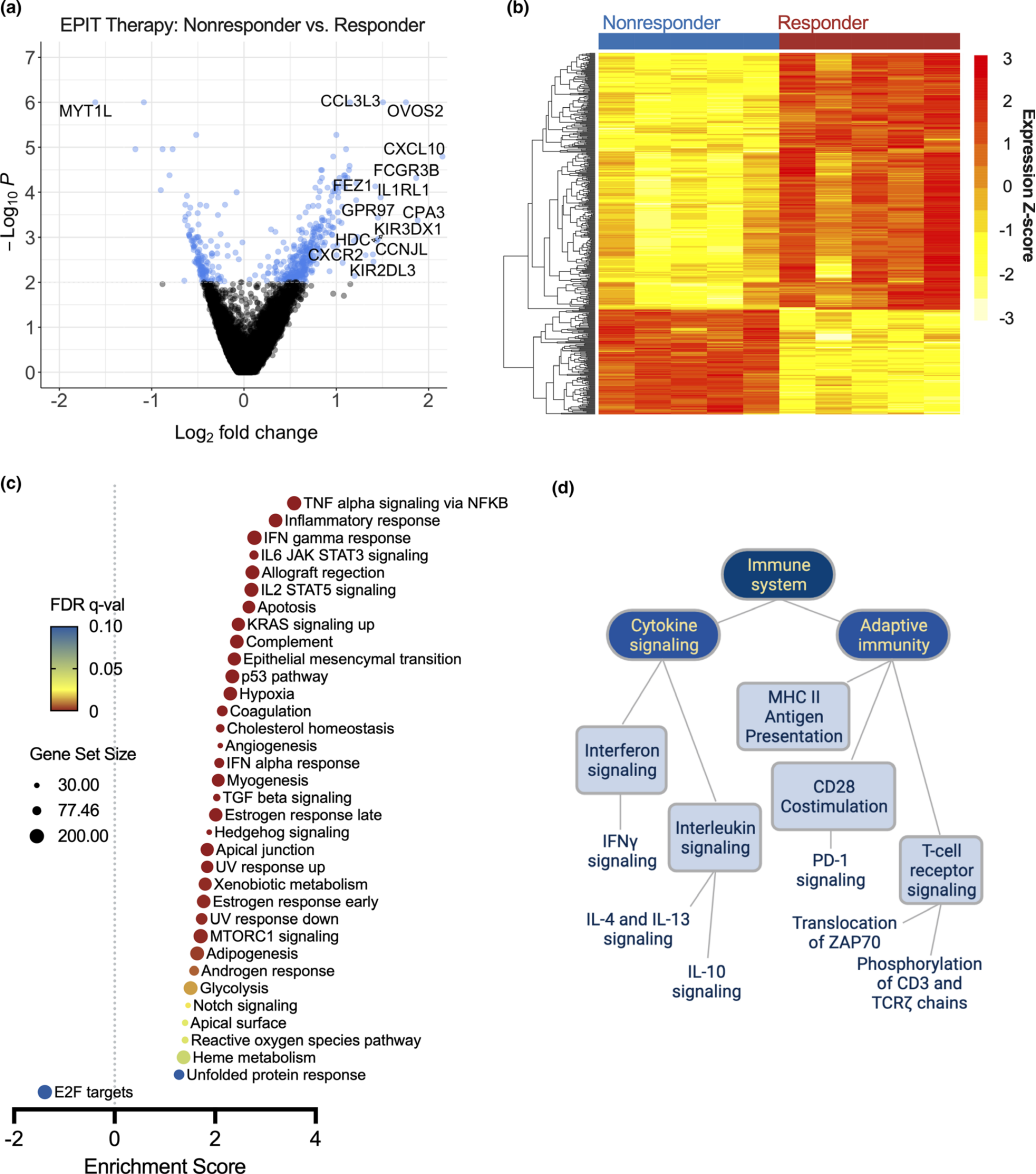
Peripheral CD4^+^ differential gene expression suggests several mechanisms may play a role in CD4^+^‐mediated EPIT responses. Differentially expressed genes (DEGs) from peripheral CD4^+^ T cells of EoE patients with favorable (responders, *n* = 5, post‐EPIT biopsy < 15 eos hpf^−1^) vs unfavorable (nonresponders, *n* = 6, post‐EPIT biopsy ≥ 15 eos hpf^−1^) clinical response to EPIT represented as: **(a)** volcano plot showing *P*‐value (Log_10_) versus fold change expression (Log_2_) with points *P* ≤ 0.01 graphed in blue; **(b)** heatmap of DEGs at *P* ≤ 0.01, with row‐wise *Z*‐score mapping as shown in the legend. **(c)** Hallmark GSEA from RNA‐Seq experiments from responder vs. nonresponder DEGs. Results with FDR *q*‐val < 0.1 are plotted by normalised enrichment score (NES), dot size represents the number of genes in the pathway, and colour represents the GSEA FDR as represented in the legend. **(d)** REACTOME pathway analysis of DEGs (FDR ≤ 0.1) from the comparison of EPIT responder to nonresponder groups. Pathways with FDR < 0.1 are shown.

Within our patient cohort, five patients that were randomised to EPIT had final biopsy results with resolution of mucosal inflammation during the milk challenge (Figure 1c, responder group; mean eos hpf^−1^ 6.0 ± 5.1), whereas six patients continued to have active mucosal inflammation despite receiving active EPIT therapy (Figure 1c, nonresponder group, mean eos hpf^−1^ ± SD, 80.2 ± 37.6). Using cut‐off of *P* ≤ 0.01, we find 470 DEGs in the comparison of EPIT responder to nonresponder patient samples (Figure 4a), with 334 upregulated transcripts and 136 downregulated transcripts (Figure 4b, heatmap). We next performed ranked GSEA using MSigDB Hallmark gene set collection to examine enriched pathways in the DEGs in the comparison of patients with favorable clinical response to EPIT versus those on EPIT therapy without clinical response (Figure 4c, GSEA ‘responders’ vs ‘nonresponders’). In this analysis, we observe enrichment of ‘TNFα signalling via NF‐κB’, IFN‐γ and IFN‐α response pathways, and ‘Epithelial mesenchymal transition’, and ‘IL6 JAK STAT3 signalling’ pathways, similarly to that seen in the on‐ vs off‐milk and EPIT vs placebo comparisons. Here, we observe negative enrichment in the ‘E2F targets pathway’, whereas downregulation of Myc targets was represented in two pathways in EPIT versus placebo comparison (Figure 3b).

We performed additional pathway analysis comparing therapy responders to nonresponders to more thoroughly examine the gene signature associated with a favorable response to therapy. We used the REACTOME database to identify possible pathways implicated in CD4^+^ response to EPIT and explore how these pathways may interconnect mechanistically (Figure 4d, Supplementary table 5). Broadly, these pathways relate to several categories of immune function: T‐cell receptor signalling (*P* = 1.16 × 10^−14^), antigen presentation and costimulation, and cytokine signalling (*P* = 1.11 × 10^−16^), including interferon gamma signalling (*P* = 1.11 × 10^−16^). Interleukin (IL)‐4 and IL‐13 signalling pathways were also identified as distinguishing EPIT responders from nonresponders, which corresponds to our previous data demonstrating that systemic antigen‐specific Th2 cells could be detected in the peripheral circulation of EoE patients during active EoE disease.^16^

We next set out to examine the potential contributions of specific T‐cell subsets to the peripheral CD4^+^ EPIT response. To do so, we leveraged data from purified human T‐cell populations^25^, ^26^, ^27^, ^28^, ^29^ to generate gene expression signatures unique to CD4^+^ helper T‐cell and regulatory T‐cell populations. We identified a set of unique DEGs for Th1, Th2 and Treg cell types, then compared our sample's average differential expression compared to these CD4^+^ subset‐specific gene groups. Overall, our analysis demonstrates a higher average expression of Th2‐specific subset genes in the ‘on‐ > off‐milk’ comparison when compared to the ‘EPIT > placebo’ comparison (Supplementary figure 3a). Post‐EPIT, however, we observe higher expression corresponding with Th1‐specific genes. This suggests that within our unsorted CD4^+^ T‐cell population there may be shifts in gene expression or cell number favoring Th1 type cells post‐EPIT. Similarly, we applied this analysis using a signature from a purified human CD4^+^CD25^hi^CD127^low^ regulatory T‐cell population as a comparator (Supplementary figure 3b). Peripheral CD4^+^ from EPIT‐treated patients have a higher per cent average expression of genes specific for regulatory T cells.

### Correlation of peripheral T‐cell gene expression to EoE transcriptome

The mucosal tissue in EoE is characterised by an eosinophil‐rich, dense inflammatory infiltrate and reactive epithelial changes. Biopsy tissue from patients with EoE has reproducible alterations in gene expression which are collectively known as the EoE transcriptome.^30^, ^31^ Rochman and colleagues have previously demonstrated that 35–40% of these transcripts arise uniquely from the oesophageal epithelium; however, many of the dysregulated transcripts within EoE biopsies can likely be attributed to hematopoietic cell types.

**Figure 5:**
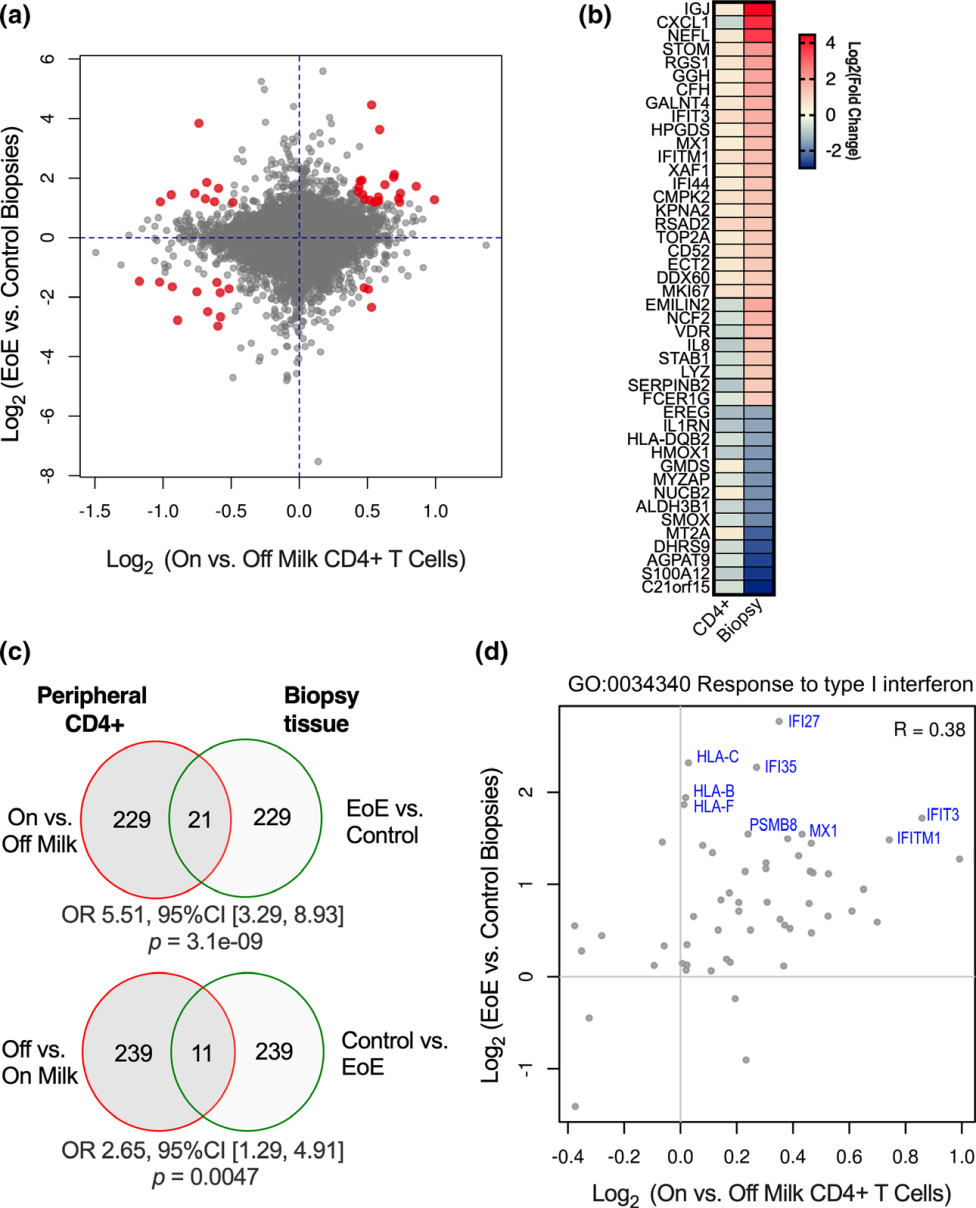
Differentially expressed genes from peripheral CD4^+^ T cells and from EoE‐specific oesophageal biopsy transcriptome. **(a)** Fold change overlap of differentially expressed genes from EoE patient peripheral CD4^+^ samples (on and off of milk‐containing diet) compared to RNA‐seq dataset from Sherrill *et al* (EoE vs. control biopsy).^30^ The top 250 genes with the highest fold change and *P* ≤ 0.01 were analysed to determine degree of overlap and are shown in red. **(b)** Heatmap display of Log_2_(fold change) of 44 overlapping genes from on‐ vs. off‐milk peripheral CD4^+^ samples and EoE vs control biopsies. **(c)** Venn diagram of overlapping differential gene expression (at *P* ≤ 0.01) between on‐ > off‐milk and EoE > control biopsy and off‐ > on‐milk and control > EoE biopsy. Significance determined using Fisher's exact test. Additional comparisons were not significant. **(d)** Fold change graph of the transcripts in the ‘Gene Ontology: resource type I interferon response’ pathway, annotated to display transcripts enriched in both biopsy tissue as well as CD4^+^ samples.

Using EoE transcriptome data from Sherrill *et al*.,^30^ we compared genes uniquely expressed in oesophageal tissue during active EoE to our on‐ versus off‐milk CD4^+^ DEGs. Our goal was to determine which alterations in peripheral CD4^+^ signalling may have the most physiologic relevance to the mucosal disease seen in EoE patients. These data sets had 14 281 shared transcripts available for analysis, from which we analysed the overlap of the top 250 genes with *P* ≤ 0.01 and highest fold change (Figure 5a). Within this subset of overlapping genes, the magnitude of the fold change in expression is higher between EoE and control biopsy tissue than it is in the on‐ versus off‐milk CD4^+^ samples (Figure 5b). Among the DEGs, there is a significant correlation between transcripts upregulated in inflamed EoE biopsies (EoE > control) and in the on‐ versus off‐milk EoE patient samples (Figure 5c; OR 5.51 95% CI [3.29, 8.93], *P* = 3.1e‐09). There also significant overlap between the off‐ > on‐milk and control > EoE comparison (Figure 5c; OR 2.67 95% CI [1.29, 4.29], *P* = 0.0047). Other comparisons were not significant.

We hypothesised that gene expression changes in the peripheral CD4^+^ T‐cell compartment of EoE patients on milk‐containing diets (i.e. on‐ > off‐milk DEGs) may correlate with EoE disease activity through T‐cell‐mediated mucosal mechanisms. To examine this hypothesis, we performed over‐representation analysis using the overlapping set of expressed genes to determine possible mechanistic pathways. Results with *P*‐values ≤ 0.01 in both the EoE versus control biopsy and on‐milk versus off‐milk SMILEE peripheral CD4^+^ samples demonstrated several overlapping pathways between the on‐ > off‐CD4^+^ T cells and EoE biopsy transcriptome (Supplementary table 6). Of particular interest, we observe genes in the IFN response signature pathway are upregulated in both the EoE biopsy tissue as well as the bulk CD4^+^ RNA‐seq from EoE patients during active disease (GO:0030198, Figure 5d). Specifically, this includes upregulation of IFN‐inducible genes and HLA‐class I molecules in both samples. In contrast, other overlapping pathways (Supplementary figure 4) involving expression of other cytokine and histone genes are not as clearly correlated between the peripheral blood CD4^+^ and mucosa during active disease.

## Discussion

In this study, we examined the gene expression profiles of peripheral CD4^+^ cells in eosinophilic oesophagitis patients. We identify specific gene expression changes that occur during active EoE in patients on a milk‐containing diet, as well as those who have completed therapy with milk antigen EPIT. In this small cohort of patients, we observe relative homogeneity in the peripheral CD4^+^ gene expression profile of active EoE patients (on‐milk) compared to inactive patients (off‐milk).

We identify IFN‐α and IFN‐γ response signatures as prominent pathways represented in GSEA from CD4^+^ DEGs of active EoE patients on milk‐containing diet when compared to nonmilk containing, inactive state. We have previously identified that EoE patients have milk antigen‐specific circulating T cells.^16^, ^24^ These antigen‐specific T‐cell populations are not present in healthy control patients, and although the numbers of these cells are reduced during periods of inactive EoE, they remain detectable using *in vitro* assays.^16^ We have recently shown that an IFN‐α and IFN‐γ gene response signature is consistently seen in both paediatric and adult EoE biopsy tissue.^24^ In this current analysis, we identify upregulated IFN responses as a distinguishing feature in unstimulated peripheral CD4^+^ cells from patients with EoE patients. Our prior work has additionally shown that EoE patients have a population of milk antigen responsive peripheral CD4^+^IFNγ T cells. Although EoE is known to have a reproducible pattern of mucosal Th2 cytokine elevation,^14^, ^32^ our results suggest altered IFN responses are present both in the mucosa and in the circulating CD4^+^ T cells of EoE patients and may be another important feature of the inflammatory milieu in these patients.

We selected the cut‐off of *P* ≤ 0.01 for our differential gene expression analysis because our primary goal was to perform ranked gene set enrichment analyses. This strategy would allow us to identify potential pathways or mechanisms of interest in peripheral CD4^+^ T‐cell responses before and after EPIT therapy in EoE patients. From these pathway analyses, we chose an FDR cut‐off of 0.1 for downstream pathway analyses to balance the risk of false‐positive results given the sample size and heterogeneity. One strength of our current study is that in the on‐ and off‐milk comparison we had a high proportion of paired samples (Supplementary table 1), reducing the impact of interindividual variability present in cross‐sectional studies. The determination of enriched pathways in the CD4^+^ cells isolated from these patient groups allowed us to find gene sets that may help identify genes/proteins with important biological relevance to the pathogenesis of EoE as well as therapeutic response to EPIT. Focusing on single genes alone would have missed such pathways and subnetworks.

In the pathway analysis from on‐ vs off‐milk, placebo vs EPIT, and responder vs nonresponder conditions, we observe enrichment in sex steroid response pathways. This cohort is 68% male, which is consistent with US cohorts and reflects the male predilection described in EoE. However, because our cohort was small and the number of patients of each sex was unevenly distributed, we did not consider Y chromosome genes in our analysis. Therefore, these findings require validation in a larger cohort where more detailed analyses stratified by sex could be performed.

We performed exploratory analyses to determine whether transcriptional changes following EPIT could be correlated with gene expression signatures from purified T‐cell subsets (Supplementary figure 3). Following EPIT, there is a decrease in the average expression of Th2‐associated transcripts, while the average expression of regulatory T‐cell‐associated transcripts increases. An imbalance of Th2 and Th1 T‐cell responses has been shown to play a critical role in allergic disease, including but not limited to EoE.^13^, ^16^, ^33^ Altering the balance between these Th2 and Th1 responses is one of several important mechanisms invoked by other successful immunotherapy strategies.^34^ We observed an increase in the average gene expression of transcripts specific to CD4^+^CD25^hi^CD127^low^ T_reg_ cells (Supplementary figure 3b). These findings are consistent with our REACTOME pathway analyses (Figure 4d) identifying aspects of Th2 signalling (i.e. IL‐4 signalling, IL‐13 signalling), regulatory T‐cell pathways (IL‐10, TGFβ and activin/follistatin), in addition to TCR and costimulatory pathway signalling, as gene expression pathways that differ in patients with a favorable clinical response to EPIT versus those without.^35^ In preclinical studies, increased numbers of gastrointestinal‐homing LAP^+^FoxP3^−^ T_reg_ cells were observed following EPIT.^21^, ^36^ However, because our experiments examine RNA‐seq signatures from the polyclonal peripheral CD4^+^ population, we cannot establish whether the observed changes are attributable to altered numbers of T‐cell subsets or because of significant transcriptional changes in a relatively constant proportion of T‐cell subsets.

The SMILEE trial was a small phase IIa trial (*n* = 20 patients).^17^ Patients following the predefined EPIT protocol had significant biopsy improvement (25.57 ± 31.19 eos hpf^−1^) compared to the placebo (95 ± 63.64 eos hpf^−1^).^17^ Limitations to our study include protocol violations that occurred during the SMILEE study (Table 1). An additional caveat to our findings is that all patients within the SMILEE study had similar histories of atopic comorbidity but this was not specifically matched in patients and controls. It is possible that antigen‐specific responses to EPIT therapy, as well as the transcriptional differences that we observe could be different in patients with a different disease phenotype. Recent studies illustrate differing biopsy transcriptional profiles are one feature of potential EoE endotypes,^32^, ^37^ and it is unclear whether peripheral lymphocyte gene expression could differ based on endotype as well. Because we assessed gene expression at the time of oesophageal biopsy, one strength of our study is that we were able to clearly correlate peripheral CD4^+^ gene expression changes with EoE disease activity.

EoE has a unique non‐IgE‐mediated pathophysiology that has previously limited the use of food antigen immunotherapy strategies for its treatment. However, the SMILEE trial has been the first of its kind to demonstrate initial promise using an immunotherapy approach to treat patients with EoE. Following a total 22‐month open‐label extension of the SMILEE trial, 47% of patients tolerated a milk reintroduction challenge without any recrudescence of oesophageal eosinophilia. In conjunction with our prior work that demonstrates antigen‐specific CD4^+^ T cells are present in the peripheral blood of EoE patients,^16^ we now show that active inflammation in EoE is associated with global alteration in CD4^+^ gene expression. A distinct feature of CD4^+^ gene expression in EoE patients is IFN‐α and IFN‐γ response signatures. This suggests that the T‐cell population we have seen during *in vitro* milk antigen stimulation followed by flow cytometry^24^ is present in patients and may play a role in EoE pathophysiology. Further, milk EPIT in EoE patients alters a subset of transcripts within the overall CD4^+^ T‐cell population, suggesting that further examination of peripheral CD4^+^ T‐cell responses in EoE is necessary to dissect the specific populations implicated in patients' response to EPIT and understand how to implement this therapy more effectively for EoE patients.

## Methods

### Study subjects

This additional research investigation and the SMILEE (Study Efficacy and Safety of the Viaskin Milk for Treating Milk Induced Eosinophilic Esophagitis in Children) trial were approved by the Institutional Review Board of the Children's Hospital of Philadelphia. All procedures were in accordance with the ethical standards of the committee and the 1964 Declaration of Helsinki and its amendments. Written informed consent was obtained from all participants for participation in the SMILEE clinical trial, as well as for this separate investigational study as an independent consideration. Full results from the SMILEE double‐blind, placebo‐controlled, phase IIa safety and efficacy study of Viaskin Milk have been published elsewhere (DBV Technologies, Bagneux, France; ClinicalTrials.gov, NCT02579876).^17^

Peripheral blood samples were collected from the subset of SMILEE patients electing to participate in this additional, optional, research study (Table 1). Briefly, patients were eligible to enrol in the SMILEE trial if they were between the ages of 4 and 17 years with a history of confirmed EoE diagnosis defined as oesophageal symptoms consistent with EoE and history of prior biopsy with > 15 eosinophils per high‐powered field (eos hpf^−1^) following a minimum 2‐month period of high‐dose proton pump inhibitor (PPI, i.e. 1–2 mg kg^−1^ omeprazole twice daily). To ensure that cow's milk was a relevant EoE antigen for each patient, screening endoscopies were performed prior to randomisation in the SMILEE study (Figure 1, schematic). Subjects underwent endoscopy while eating a cow's milk‐containing diet (minimum of 240 mL milk per day) for 8 weeks minimum. A second endoscopy was performed following 8 weeks of a milk‐free diet. If biopsies confirmed milk‐responsive EoE (> 15 eos hpf^−1^ on milk‐containing diet and < 10 eos hpf^−1^ on milk‐free diet), patients were eligible for randomisation in the SMILEE trial with Viaskin Milk (500 µg of milk proteins) or placebo patch (Figure 1, DBV Technologies, Paris, France).

Following 9 months of EPIT while on a milk‐free diet, EPIT was continued throughout a 2‐month period of milk reintroduction (minimum 240 mL milk daily). Patients then underwent repeat endoscopy with biopsy to assess response to therapy. Patients randomised to the active (milk peptide Viaskin EPIT) treatment arm were further classified as treatment responders or nonresponders based on these post‐EPIT biopsy results. Patients were classified as therapy ‘responders’ if post‐EPIT biopsy was < 15 eos hpf^−1^ and ‘nonresponders’ if post‐EPIT biopsy continued to have significant inflammation with > 15 eos hpf^−1^.

### CD4^+^ T‐cell isolation

Peripheral blood samples were collected at each SMILEE study endoscopy (‘on milk‐containing diet’ screening visit, ‘off‐milk‐containing diet’ screening visit and following EPIT therapy, Figure 1). Whole‐blood samples were stored at 4°C for less than 4 h before PBMCs were separated by density gradient centrifugation (Ficoll‐Paque Plus, GE Healthcare Lifesciences, Marlborough, MA, USA) and cultured on a polystyrene flask for 1 h to remove remaining adherent cells. CD4^+^ cells were isolated via positive selection using magnetic beads (Invitrogen Dynabeads Human CD4^+^ Positive Isolation Kit, Carlsbad, CA, USA), which achieved a median purity of 95% CD4^+^ T cells confirmed by flow cytometry analysis.

### RNA isolation and sequencing

Total CD4^+^ T‐cell RNA was extracted (TRIzol reagent, Invitrogen, Carlsbad, CA, USA) and purified (RNeasy Mini Kit, Qiagen, Hilden, Germany). RNA preparations with an A260/280 range of 1.8–2.1 and RNA integrity number over 7 were used for further analysis. cDNA was generated and libraries were made with Ovation Ultralow Library System V2 (NuGen, Redwood City, CA, USA), samples were pooled, and paired‐end reads were generated using an Illumina HiSeq 4000 (Illumina, San Diego, CA, USA) with a minimum of 50 × 10^6^ confirmed reads per library.

### Analysis of RNA‐Seq data

Sequence reads were aligned to the human reference genome GRCh38 and transcriptome using STAR.^38^, ^39^ Gene‐level read counts (in FPKM) were normalised using the Rlog method and log_2_‐transformed.^40^ Genes with total read counts < 6 were removed from analysis. As a result of unequal distribution of patients of different sexes between groups, Y chromosome genes were not included in the analysis. Reads mapping to both strands of transcripts were counted to account for double‐stranded libraries. Differential gene expression between treatment groups was tested using the rank products method,^41^ which is a robust nonparametric test suitable to highly variable clinical samples. The data from this publication have been deposited in NCBI's Gene Expression Omnibus (https://www.ncbi.nlm.nih.gov/geo/) at accession number GSE173895.

### Gene set enrichment and pathway analysis

Genes were ranked by their *P*‐values and direction of change and input into gene set enrichment analysis (GSEA) algorithms.^42^, ^43^ Hallmark gene sets from the molecular signatures database were used to identify pathways significantly enriched with differentially expressed genes (DEGs).^44^ Pathways with an FDR < 0.1 were selected for comparison between on‐milk, off‐milk and post‐therapy comparisons. Reactome pathway analysis was performed for genes in the responder vs nonresponder comparison using DEGs meeting FDR < 0.1 cut‐off, and REACTOME pathways with FDR < 0.1 are reported.^45^, ^46^

### Comparative analyses

Data were downloaded from Gene Expression Omnibus (GEO, http://www.ncbi.nlm.nih.gov/geo/) to perform comparative analyses.^25^, ^26^, ^27^, ^28^, ^29^, ^47^ Purified, unstimulated, human T‐cell subset gene expression data (GSE3982 and GSE107011) were used to determine difference in average gene expression from our patients' CD4^+^ samples compared to Th1, Th2 and regulatory T‐cell subsets. Published datasets were analysed to derive unique gene expression signatures for regulatory (GSE107011) and Th1/Th2 (GSE3982) CD4^+^ T‐cell subsets.^28^, ^29^, ^47^ For each GEO data set, ANOVA was used to identify genes differentially expressed between all T‐cell subtypes and Limma was used to identify unique differentially expressed genes for Th1, Th2 and Treg cell types. Then, each patient sample was compared to this T‐cell subset data. We calculated per cent change of all genes in our samples across the specific comparisons (on‐ vs off‐milk and EPIT vs placebo comparisons) for the sets of Th1, Th2 and regulatory CD4^+^ T‐cell genes identified.

EoE mucosal tissue is characterised by a reproducible set of transcriptional changes, as demonstrated by Sherril and colleagues using RNA sequencing of biopsy tissue from EoE patients and controls (GSE58640).^25^, ^26^, ^30^ Sample CD4^+^ data were compared to this dataset to examine potential overlapping signatures of biologic importance in EoE, using ANOVA to identify genes expressed between both datasets.

## Conflict of interest

Drs Spergel and Cianferoni have consulting agreements and clinical trial grants with DBV Technologies. Dr Spergel has stock equity with DBV Technologies. DBV Technologies provided the Viaskin Patch used in the SMILEE clinical trial and had no role in the collection, analysis or interpretation of the data presented in the current study. The remaining authors declare no competing financial interests or relationships pertinent to the work reported in this paper.

## Author contributions

**Melanie A Ruffner:** Conceptualization; Data curation; Formal analysis; Funding acquisition; Investigation; Methodology; Supervision; Validation; Visualization; Writing‐original draft; Writing‐review & editing. **Zhe Zhang:** Data curation; Formal analysis; Methodology; Visualization; Writing‐review & editing. **Kelly Maurer:** Investigation; Methodology; Project administration. **Amanda B Muir:** Methodology; Project administration; Resources. **Antonella Cianferoni:** Project administration; Resources; Writing‐review & editing. **Kathleen E Sullivan:** Conceptualization; Formal analysis; Methodology; Resources; Supervision; Validation; Visualization; Writing‐review & editing. **Jonathan M Spergel:** Conceptualization; Funding acquisition; Methodology; Project administration; Resources; Supervision; Visualization; Writing‐review & editing.

## References

[1] Dellon ES , Hirano I . Epidemiology and natural history of eosinophilic esophagitis. Gastroenterology 2018; 154: 319–332.e3. 28774845 10.1053/j.gastro.2017.06.067 PMC5794619

[2] Hommeida S , Grothe RM , Hafed Y *et al*. Assessing the incidence trend and characteristics of eosinophilic esophagitis in children in Olmsted County, Minnesota. Dis Esophagus 2018; 31: 1–8. 10.1093/dote/doy062 PMC6279968 29982568

[3] Jensen ET , Kappelman MD , Martin CF , Dellon ES . Health‐care utilization, costs, and the burden of disease related to eosinophilic esophagitis in the United States. Am J Gastroenterol 2015; 110: 626–632. 25267327 10.1038/ajg.2014.316 PMC4590991

[4] Ruffner MA , Brown‐Whitehorn TF , Verma R *et al*. Clinical tolerance in eosinophilic esophagitis. J Allergy Clin Immunol Pract 2018; 6: 661–663. 28811175 10.1016/j.jaip.2017.06.035 PMC5809321

[5] Jensen ET , Kappelman MD , Kim HP , Ringel‐Kulka T , Dellon ES . Early life exposures as risk factors for pediatric eosinophilic esophagitis. J Pediatr Gastroenterol Nutr 2013; 57: 67–71. 23518485 10.1097/MPG.0b013e318290d15a

[6] Jensen ET , Kuhl JT , Martin LJ , Langefeld CD , Dellon ES , Rothenberg ME . Early‐life environmental exposures interact with genetic susceptibility variants in pediatric patients with eosinophilic esophagitis. J Allergy Clin Immunol 2017; 141: 632–637. 29029802 10.1016/j.jaci.2017.07.010 PMC5803324

[7] Sleiman PMA , Wang M‐L , Cianferoni A *et al*. GWAS identifies four novel eosinophilic esophagitis loci. Nat Commun 2014; 5: 5593. 25407941 10.1038/ncomms6593 PMC4238044

[8] Kottyan LC , Davis BP , Sherrill JD *et al*. Genome‐wide association analysis of eosinophilic esophagitis provides insight into the tissue specificity of this allergic disease. Nat Genet 2014; 46: 895–900. 25017104 10.1038/ng.3033 PMC4121957

[9] Kelly KJ , Lazenby AJ , Rowe PC , Yardley JH , Perman JA , Sampson HA . Eosinophilic esophagitis attributed to gastroesophageal reflux: improvement with an amino acid‐based formula. Gastroenterology 1995; 109: 1503–1512. 7557132 10.1016/0016-5085(95)90637-1

[10] Milner JD , Ward JM , Keane‐Myers A , Paul WE . Lymphopenic mice reconstituted with limited repertoire T cells develop severe, multiorgan, Th2‐associated inflammatory disease. Proc Natl Acad Sci USA 2007; 104: 576–581. 17202252 10.1073/pnas.0610289104 PMC1761908

[11] Mishra A , Schlotman J , Wang M , Rothenberg ME . Critical role for adaptive T cell immunity in experimental eosinophilic esophagitis in mice. J Leukoc Biol 2007; 81: 916–924. 17194734 10.1189/jlb.1106653

[12] Straumann A , Bauer M , Fischer B , Blaser K , Simon H‐U . Idiopathic eosinophilic esophagitis is associated with a TH2‐type allergic inflammatory response. J Allergy Clin Immunol 2001; 108: 954–961. 11742273 10.1067/mai.2001.119917

[13] Wen T , Aronow BJ , Rochman Y *et al*. Single‐cell RNA sequencing identifies inflammatory tissue T cells in eosinophilic esophagitis. J Clin Invest 2019; 129: 2014–2028. 30958799 10.1172/JCI125917 PMC6486341

[14] Blanchard C , Stucke EM , Rodriguez‐Jimenez B *et al*. A striking local esophageal cytokine expression profile in eosinophilic esophagitis. J Allergy Clin Immunol 2011; 127: 208–217.e7. 21211656 10.1016/j.jaci.2010.10.039 PMC3027004

[15] Bullock JZ , Villanueva JM , Blanchard C *et al*. Interplay of adaptive Th2 immunity with eotaxin‐3/C‐C chemokine receptor 3 in eosinophilic esophagitis. J Pediatr Gastroenterol Nutr 2007; 45: 22–31. 17592361 10.1097/MPG.0b013e318043c097

[16] Cianferoni A , Ruffner MA , Guzek R *et al*. Elevated expression of activated TH2 cells and milk‐specific TH2 cells in milk‐induced eosinophilic esophagitis. Ann Allergy Asthma Immunol 2018; 120: 177–183.e2. 29289462 10.1016/j.anai.2017.11.006 PMC5875940

[17] Spergel JM , Elci OU , Muir AB *et al*. Efficacy of epicutaneous immunotherapy in children with milk‐induced eosinophilic esophagitis. Clin Gastroenterol Hepatol 2020; 18: 328–336.e7. 31100455 10.1016/j.cgh.2019.05.014

[18] Spergel JM , Brown‐Whitehorn TF , Beausoleil JL *et al*. 14 years of eosinophilic esophagitis: clinical features and prognosis. J Pediatr Gastroenterol Nutr 2009; 48: 30–36. 19172120 10.1097/MPG.0b013e3181788282

[19] Dupont C , Kalach N , Soulaines P , Legoué‐Morillon S , Piloquet HBP . Cow's milk epicutaneous immunotherapy in children: a pilot trial of safety, acceptability, and impact on allergic reactivity. J Allergy Clin Immunol 2010; 125: 1165–1167. 20451043 10.1016/j.jaci.2010.02.029

[20] Fleischer DM , Greenhawt M , Sussman G *et al*. Effect of epicutaneous immunotherapy vs placebo on reaction to peanut protein ingestion among children with peanut allergy. JAMA 2019; 321: 946. 30794314 10.1001/jama.2019.1113 PMC6439674

[21] Mondoulet L , Dioszeghy V , Larcher T *et al*. Epicutaneous immunotherapy (EPIT) blocks the allergic esophago‐gastro‐enteropathy induced by sustained oral exposure to peanuts in sensitized mice. PLoS One 2012; 7: e31967. 22363776 10.1371/journal.pone.0031967 PMC3283696

[22] Mondoulet L , Dioszeghy V , Busato F *et al*. *Gata3* hypermethylation and *Foxp3* hypomethylation are associated with sustained protection and bystander effect following epicutaneous immunotherapy in peanut‐sensitized mice. Allergy 2019; 74: 152–164. 29779209 10.1111/all.13479 PMC6585762

[23] Dioszeghy V , Mondoulet L , Laoubi L *et al*. Antigen uptake by langerhans cells is required for the induction of regulatory T cells and the acquisition of tolerance during epicutaneous immunotherapy in OVA‐sensitized mice. Front Immunol 2018; 9: 1951. 30233572 10.3389/fimmu.2018.01951 PMC6129590

[24] Ruffner MA , Hu A , Dilollo J *et al*. Conserved IFN signature between adult and pediatric eosinophilic esophagitis. J Immunol 2021; 206: 1361–1371. 33558373 10.4049/jimmunol.2000973 PMC7946729

[25] Barrett T , Wilhite SE , Ledoux P *et al*. NCBI GEO: archive for functional genomics data sets—update. Nucleic Acids Res 2012; 41: D991–D995. 23193258 10.1093/nar/gks1193 PMC3531084

[26] Edgar R , Domrachev M , Lash AE . Gene expression omnibus: NCBI gene expression and hybridization array data repository. Nucleic Acids Res 2002; 30: 207–210. 11752295 10.1093/nar/30.1.207 PMC99122

[27] Jeffrey KL , Brummer T , Rolph MS *et al*. Positive regulation of immune cell function and inflammatory responses by phosphatase PAC‐1. Nat Immunol 2006; 7: 274–283. 16474395 10.1038/ni1310

[28] Monaco G , Lee B , Xu W *et al*. RNA‐Seq signatures normalized by mRNA abundance allow absolute deconvolution of human immune cell types. Cell Rep 2019; 26: 1627–1640.e7. 30726743 10.1016/j.celrep.2019.01.041 PMC6367568

[29] Xu W , Monaco G , Wong EH *et al*. Mapping of γ/δ T cells reveals Vδ2^+^ T cells resistance to senescence. EBioMedicine 2019; 39: 44–58. 30528453 10.1016/j.ebiom.2018.11.053 PMC6354624

[30] Sherrill JD , Kc K , Blanchard C *et al*. Analysis and expansion of the eosinophilic esophagitis transcriptome by RNA sequencing. Genes Immun 2014; 15: 361–369. 24920534 10.1038/gene.2014.27 PMC4156528

[31] Blanchard C , Wang N , Stringer KF *et al*. Eotaxin‐3 and a uniquely conserved gene‐expression profile in eosinophilic esophagitis. J Clin Invest 2006; 116: 536–547. 16453027 10.1172/JCI26679 PMC1359059

[32] Dunn JLM , Shoda T , Caldwell JM *et al*. Esophageal type 2 cytokine expression heterogeneity in eosinophilic esophagitis in a multisite cohort. J Allergy Clin Immunol 2020; 145: 1629–1640.e4. 32197970 10.1016/j.jaci.2020.01.051 PMC7309223

[33] Wambre E , Bajzik V , DeLong JH *et al*. A phenotypically and functionally distinct human TH2 cell subpopulation is associated with allergic disorders. Sci Transl Med 2017; 9: eaam9171. 28768806 10.1126/scitranslmed.aam9171 PMC5987220

[34] Sampson HA , O'Mahony L , Burks AW , Plaut M , Lack G , Akdis CA . Mechanisms of food allergy. J Allergy Clin Immunol 2018; 141: 11–19. 29307410 10.1016/j.jaci.2017.11.005

[35] Huber S , Stahl FR , Schrader J *et al*. Activin A promotes the TGF‐β‐induced conversion of CD4^+^ CD25^−^ T cells into Foxp3^+^ induced regulatory T cells. J Immunol 2009; 182: 4633–4640. 19342638 10.4049/jimmunol.0803143

[36] Dioszeghy V , Mondoulet L , Dhelft V *et al*. The regulatory T cells induction by epicutaneous immunotherapy is sustained and mediates long‐term protection from eosinophilic disorders in peanut‐sensitized mice. Clin Exp Allergy J Br Soc Allergy Clin Immunol 2014; 44: 867–881. 10.1111/cea.12312 PMC4233996 24666588

[37] Shoda T , Matsuda A , Nomura I *et al*. Eosinophilic esophagitis versus proton pump inhibitor–responsive esophageal eosinophilia: transcriptome analysis. J Allergy Clin Immunol 2017; 139: 2010–2013.e4. 28063872 10.1016/j.jaci.2016.11.028

[38] Schneider VA , Graves‐Lindsay T , Howe K *et al*. Evaluation of GRCh38 and *de novo* haploid genome assemblies demonstrates the enduring quality of the reference assembly. Genome Res 2017; 27: 849–864. 28396521 10.1101/gr.213611.116 PMC5411779

[39] Dobin A , Davis CA , Schlesinger F *et al*. STAR: ultrafast universal RNA‐seq aligner. Bioinformatics 2013; 29: 15–21. 23104886 10.1093/bioinformatics/bts635 PMC3530905

[40] Love MI , Huber W , Anders S . Moderated estimation of fold change and dispersion for RNA‐seq data with DESeq2. Genome Biol 2014; 15: 550. 25516281 10.1186/s13059-014-0550-8 PMC4302049

[41] Breitling R , Armengaud P , Amtmann A , Herzyk P . Rank products: a simple, yet powerful, new method to detect differentially regulated genes in replicated microarray experiments. FEBS Lett 2004; 573: 83–92. 15327980 10.1016/j.febslet.2004.07.055

[42] Mootha VK , Lindgren CM , Eriksson K‐F *et al*. PGC‐1α‐responsive genes involved in oxidative phosphorylation are coordinately downregulated in human diabetes. Nat Genet 2003; 34: 267–273. 12808457 10.1038/ng1180

[43] Subramanian A , Tamayo P , Mootha VK *et al*. Gene set enrichment analysis: a knowledge‐based approach for interpreting genome‐wide expression profiles. Proc Natl Acad Sci USA 2005; 102: 15545–15550. 16199517 10.1073/pnas.0506580102 PMC1239896

[44] Liberzon A , Birger C , Thorvaldsdóttir H , Ghandi M , Mesirov JP , Tamayo P . The Molecular Signatures Database (MSigDB) hallmark gene set collection. Cell Syst 2015; 1: 417–425. 26771021 10.1016/j.cels.2015.12.004 PMC4707969

[45] Fabregat A , Jupe S , Matthews L *et al*. The reactome pathway knowledgebase. Nucleic Acids Res 2018; 46: D649–D655. 29145629 10.1093/nar/gkx1132 PMC5753187

[46] Sidiropoulos K , Viteri G , Sevilla C *et al*. Reactome enhanced pathway visualization. Bioinformatics 2017; 33: 3461–3467. 29077811 10.1093/bioinformatics/btx441 PMC5860170

[47] Chtanova T , Tangye SG , Newton R *et al*. T follicular helper cells express a distinctive transcriptional profile, reflecting their role as non‐Th1/Th2 effector cells that provide help for B cells. J Immunol 2004; 173: 68–78. 15210760 10.4049/jimmunol.173.1.68

