# Supplementary material for: RNA sequencing identifies global transcriptional changes in peripheral CD4+ cells during active oesophagitis and following epicutaneous immunotherapy in eosinophilic oesophagitis
Source: Clin Transl Immunology. 2021 Jul 22;10(7):e1314. doi: 10.1002/cti2.1314 (PMC8296633; doi:10.1002/cti2.1314)
Supplement: Supplementary file 1 [file CTI2-10-e1314-s001.docx]

**Supplementary Table 1: Information regarding patient sample pairing for enrollment endoscopy samples.** Age, sex and biopsy results from each EoE patient before and after milk-containing diet are shown. Dashed lines indicate that there was no sample available for analysis at that timepoint.

| Age | Sex | “Off milk”  Biopsy eos/hpf | “On Milk” Biopsy eos/hpf |
| --- | --- | --- | --- |
| 11 | M | 0-7 | 134 |
| 9 | M | 0 | 75 |
| 13 | M | -- | 15 |
| 12 | M | -- | 112 |
| 8 | F | 5 | 30 |
| 12 | F | 1 | 127 |
| 5 | M | 5 | 25 |
| 6 | M | -- | 20 |
| 12 | M | -- | 30 |
| 15 | F | 3 | 25 |
| 8 | M | 6 | -- |

**Supplementary Table 2: Differentially expressed genes, EoE patients on milk containing diet (n=10 patient samples) versus off milk (n=7 patient samples) containing diet.** Using the Benjamini-Hochberg method with an FDR cutoff of 0.05, we identify 244 differentially expressed genes (DEGs) altered during milk-associated active EoE disease. Of these, 97 transcripts were upregulated and 147 were downregulated at the time of the active milk diet endoscopy. Genes with absolute fold change over 1.75 are shown in the table.

| **Gene Symbol** | **Log_2_ (Fold Change)** | **FDR** |
| --- | --- | --- |
| CXCL10 | 1.3711 | 0.0045 |
| MTRNR2L1 | 1.1504 | 0.02 |
| RSAD2 | 0.9921 | 0.0013 |
| HBB | 0.9099 | 0.0024 |
| PPIB | 0.9074 | < 10e^-4^ |
| LOC101929643 | 0.8912 | 0.034 |
| CD69 | 0.8773 | 0.0055 |
| LMOD2 | 0.865 | 0.0013 |
| IFIT3 | 0.8583 | 0.0013 |
| LAMP3 | 0.8562 | 0.0071 |
| ASB15 | 0.8434 | 0.0045 |
| LOC284581 | 0.8383 | < 10e^-4^ |
| RPS14 | 0.832 | < 10e^-4^ |
| KLRD1 | 0.831 | 0.011 |
| C1orf95 | 0.8194 | 0.009 |
| GBP1 | 0.8099 | 0.0078 |
| TMEM52B | -0.8085 | 0.01 |
| ITGAX | -0.8099 | 0.0058 |
| VCAN | -0.8103 | 0.0024 |
| THBS1 | -0.8196 | < 10e^-4^ |
| RHOB | -0.8207 | 0.0058 |
| LOC339874 | -0.8219 | 0.031 |
| C5AR1 | -0.8269 | 0.009 |
| MRAS | -0.8302 | 0.014 |
| RNASE2 | -0.8304 | 0.022 |
| CD300LF | -0.8316 | 0.025 |
| AQP9 | -0.8366 | 0.0013 |
| PPARG | -0.8373 | 0.0083 |
| PILRA | -0.846 | 0.016 |
| ADORA2B | -0.8504 | 0.009 |
| ALDH1A1 | -0.8507 | 0.0096 |
| JDP2 | -0.8534 | 0.0083 |
| LOC729737 | -0.8627 | 0.0083 |
| TREM1 | -0.8645 | 0.0058 |
| OLR1 | -0.8675 | < 10e^-4^ |
| CCDC183-AS1 | -0.8754 | 0.0013 |
| DOK3 | -0.8786 | 0.0013 |
| CLEC7A | -0.8794 | 0.0071 |
| CREB5 | -0.8801 | < 10e^-4^ |
| CCL3 | -0.8901 | < 10e^-4^ |
| S100A12 | -0.8931 | 0.0013 |
| SLC7A11-AS1 | -0.8945 | < 10e^-4^ |
| CD163 | -0.895 | < 10e^-4^ |
| BMS1P2 | -0.8982 | < 10e^-4^ |
| MMP19 | -0.9031 | < 10e^-4^ |
| BASP1 | -0.9249 | < 10e^-4^ |
| HMOX1 | -0.9321 | 0.0058 |
| *Supplementary Table 2, continued* | | |
|  |  |  |
| **Gene Symbol** | **Log_2_ (Fold Change)** | **FDR** |
| PTX3 | -0.9366 | 0.0058 |
| KCNMB1 | -0.9373 | 0.022 |
| IL8 | -0.9399 | < 10e^-4^ |
| LOC145474 | -0.9474 | 0.0024 |
| GPNMB | -0.9582 | 0.033 |
| SLC37A2 | -0.9627 | < 10e^-4^ |
| SGMS2 | -0.9733 | < 10e^-4^ |
| RPH3A | -0.979 | < 10e^-4^ |
| LIPN | -0.9833 | < 10e^-4^ |
| KYNU | -0.9839 | <10e^-4^ |
| TUBB6 | -0.9851 | 0.0013 |
| CXCL2 | -0.9903 | < 10e^-4^ |
| SERPINB2 | -1.0198 | < 10e^-4^ |
| FCAR | -1.0203 | < 10e^-4^ |
| IL1RN | -1.0253 | < 10e^-4^ |
| THBD | -1.0344 | < 10e^-4^ |
| FGD4 | -1.0453 | < 10e^-4^ |
| GAS2L3 | -1.0596 | < 10e^-4^ |
| MAFB | -1.0606 | < 10e^-4^ |
| UGDH-AS1 | -1.0607 | < 10e^-4^ |
| ANPEP | -1.1138 | < 10e^-4^ |
| TGM2 | -1.1527 | < 10e^-4^ |
| EREG | -1.1738 | < 10e^-4^ |
| MYT1L | -1.2308 | < 10e^-4^ |
| CYP1B1 | -1.2505 | < 10e^-4^ |
| CLEC5A | -1.4939 | < 10e^-4^ |

**Supplementary Table 3:** **Differentially expressed genes, EPIT therapy versus placebo therapy patients.** Using the Benjamini-Hochberg method with an FDR cutoff of 0.05, we observe a total of 129 DEGs, with 106 upregulated and 23 downregulated transcripts in CD4+ samples collected from EoE patients on EPIT (n = 7 patients) compared to those on placebo (n=3 patients). Genes with absolute fold change over 1.75 are shown in the table.

| **Gene Symbol** | **Log_2_ (Fold Change)** | **FDR** |
| --- | --- | --- |
| B3GNT7 | 1.7174 | < 10e^-4^ |
| CCL7 | 1.3058 | < 10e^-4^ |
| CXCL2 | 1.273 | < 10e^-4^ |
| CCL4 | 1.2067 | < 10e^-4^ |
| GAS2L3 | 1.2036 | < 10e^-4^ |
| CCR1 | 1.1948 | < 10e^-4^ |
| RAB7B | 1.1838 | < 10e^-4^ |
| SGMS2 | 1.1721 | < 10e^-4^ |
| MSR1 | 1.1697 | < 10e^-4^ |
| CX3CR1 | 1.1637 | 0.015 |
| EMR3 | 1.1603 | < 10e^-4^ |
| SIGLEC10 | 1.1527 | < 10e^-4^ |
| TNFSF15 | 1.1491 | < 10e^-4^ |
| LILRA1 | 1.1369 | < 10e^-4^ |
| FBN2 | 1.1353 | < 10e^-4^ |
| LOC102724696 | 1.1326 | 0.0059 |
| THBD | 1.1223 | < 10e^-4^ |
| THBS1 | 1.1209 | < 10e^-4^ |
| GPRC5A | 1.1158 | 0.0072 |
| SLC9A7P1 | 1.112 | 0.0059 |
| MAFB | 1.1102 | < 10e^-4^ |
| EMP1 | 1.0987 | < 10e^-4^ |
| SLC2A14 | 1.0959 | < 10e^-4^ |
| RHOB | 1.0815 | 0.017 |
| NID1 | 1.0738 | 0.02 |
| SERPINB2 | 1.07 | < 10e^-4^ |
| MNDA | 1.0698 | < 10e^-4^ |
| EREG | 1.0686 | 0.012 |
| SIK1 | 1.0588 | 0.0032 |
| RP11-277P12.20 | 1.0479 | 0.033 |
| FCRL1 | 1.0463 | 0.022 |
| FGD4 | 1.0133 | 0.022 |
| CD300E | 1.0132 | < 10e^-4^ |
| SIRPB1 | 1.0129 | 0.0072 |
| MS4A7 | 1.0101 | 0.03 |
| EGR2 | 1.0081 | 0.03 |
| FCAR | 1.0049 | 0.0072 |
| EGR1 | 1.0021 | 0.022 |
| SLC8A1 | 0.9953 | 0.031 |
| MYOM2 | 0.9886 | 0.034 |
| CTSW | 0.9843 | 0.03 |
| HBEGF | 0.9838 | 0.014 |
| CLEC7A | 0.9744 | 0.03 |
| FCGR3A | 0.9722 | 0.031 |
| DOCK5 | 0.966 | 0.035 |
| LOC729737 | 0.9582 | 0.0072 |
| MAP7D2 | 0.9573 | 0.025 |
| FCGR2A | 0.9556 | 0.026 |
| JDP2 | 0.9556 | 0.031 |
| KLF4 | 0.9538 | 0.0088 |
| LOC102723797 | 0.9506 | 0.041 |
| SLC44A5 | 0.9428 | < 10e^-4^ |
| MET | 0.9413 | 0.022 |
| PPARG | 0.935 | 0.02 |
| *Supplementary Table 3, continued* | | |
|  |  |  |
| **Gene Symbol** | **Log_2_ (Fold Change)** | **FDR** |
| RP11-362F19.1 | 0.9326 | 0.022 |
| FCN1 | 0.9297 | 0.022 |
| IGSF6 | 0.9291 | 0.03 |
| LYN | 0.9286 | 0.049 |
| SH2D1B | 0.9239 | 0.033 |
| MN1 | 0.922 | 0.035 |
| DUSP6 | 0.9217 | 0.022 |
| FPR1 | 0.9209 | 0.035 |
| C5AR1 | 0.9154 | 0.042 |
| FCER1A | 0.9139 | 0.0072 |
| IRAK3 | 0.9069 | 0.042 |
| AGPAT9 | 0.9056 | 0.041 |
| CSF1R | 0.9049 | 0.042 |
| CD163 | 0.9042 | 0.017 |
| EMR2 | 0.9015 | 0.043 |
| SGK1 | 0.9007 | 0.045 |
| TLR4 | 0.8971 | 0.041 |
| PTX3 | 0.8958 | 0.033 |
| CSF2RA | 0.8946 | 0.042 |
| GAS7 | 0.8942 | 0.041 |
| SLC7A11-AS1 | 0.8912 | 0.026 |
| LRRK2 | 0.8896 | 0.0088 |
| PLEK | 0.8836 | 0.042 |
| CXCL3 | 0.8812 | 0.031 |
| AQP9 | 0.8802 | 0.031 |
| ZEB2 | 0.8774 | 0.049 |
| NFIL3 | 0.8721 | 0.042 |
| PYGL | 0.8718 | 0.041 |
| FPR2 | 0.8708 | 0.049 |
| CHAC1 | 0.8681 | 0.049 |
| ANKRD20A11P | 0.8677 | 0.017 |
| RET | 0.8519 | 0.042 |
| P2RY1 | 0.8473 | 0.049 |
| SLC7A11 | 0.8445 | 0.022 |
| MGAM | 0.8405 | 0.045 |
| CXCL1 | 0.8344 | 0.022 |
| LAMA2 | 0.8255 | 0.041 |
| EGR3 | 0.8217 | 0.042 |
| CREB5 | 0.8183 | 0.022 |
| COL18A1 | -0.8213 | 0.031 |
| S1PR4 | -0.842 | 0.045 |
| LOC101929796 | -0.8462 | 0.041 |
| LIMS2 | -0.863 | 0.034 |
| TMIGD2 | -0.8762 | 0.031 |
| DBH-AS1 | -0.8766 | 0.031 |
| SRGAP2D | -0.8809 | 0.022 |
| RPPH1 | -0.9554 | 0.038 |
| LINC00997 | -1.0514 | 0.011 |
| ADARB2 | -1.0957 | 0.0088 |
| CMSS1 | -1.1218 | 0.017 |
| LOC284581 | -1.1842 | 0.0072 |
| SLC16A14 | -2.2291 | 0.012 |

**
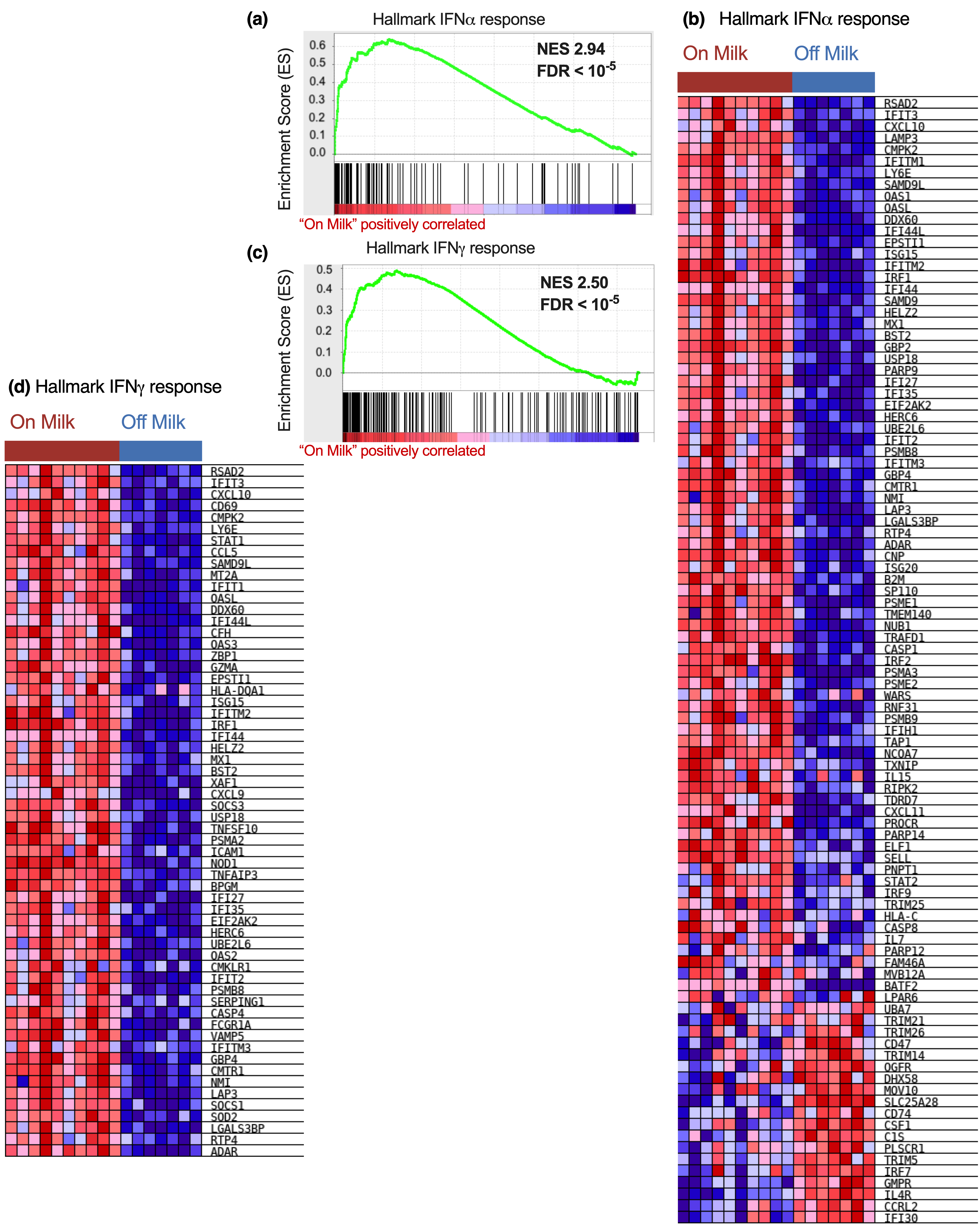
**

**Supplementary Figure 1: Prominent IFN response gene signature in CD4+ cells from EoE patients on milk-containing diet.** Gene set enrichment analysis (GSEA) was performed with the canonical pathway gene sets in GSEA Molecular Signatures Database using On versus Off milk EoE patient samples (n=10 and n=7). (A) GSEA Enrichment plot (score curves) for Hallmark IFN alpha pathway and (B) the heatmap panel visualizes all genes contributing to the enriched pathway. (C) GSEA Enrichment plot (score curves for Hallmark IFN gamma pathway and (D) the heatmap visualizes the top 60 genes contributing to the enriched pathway.

**
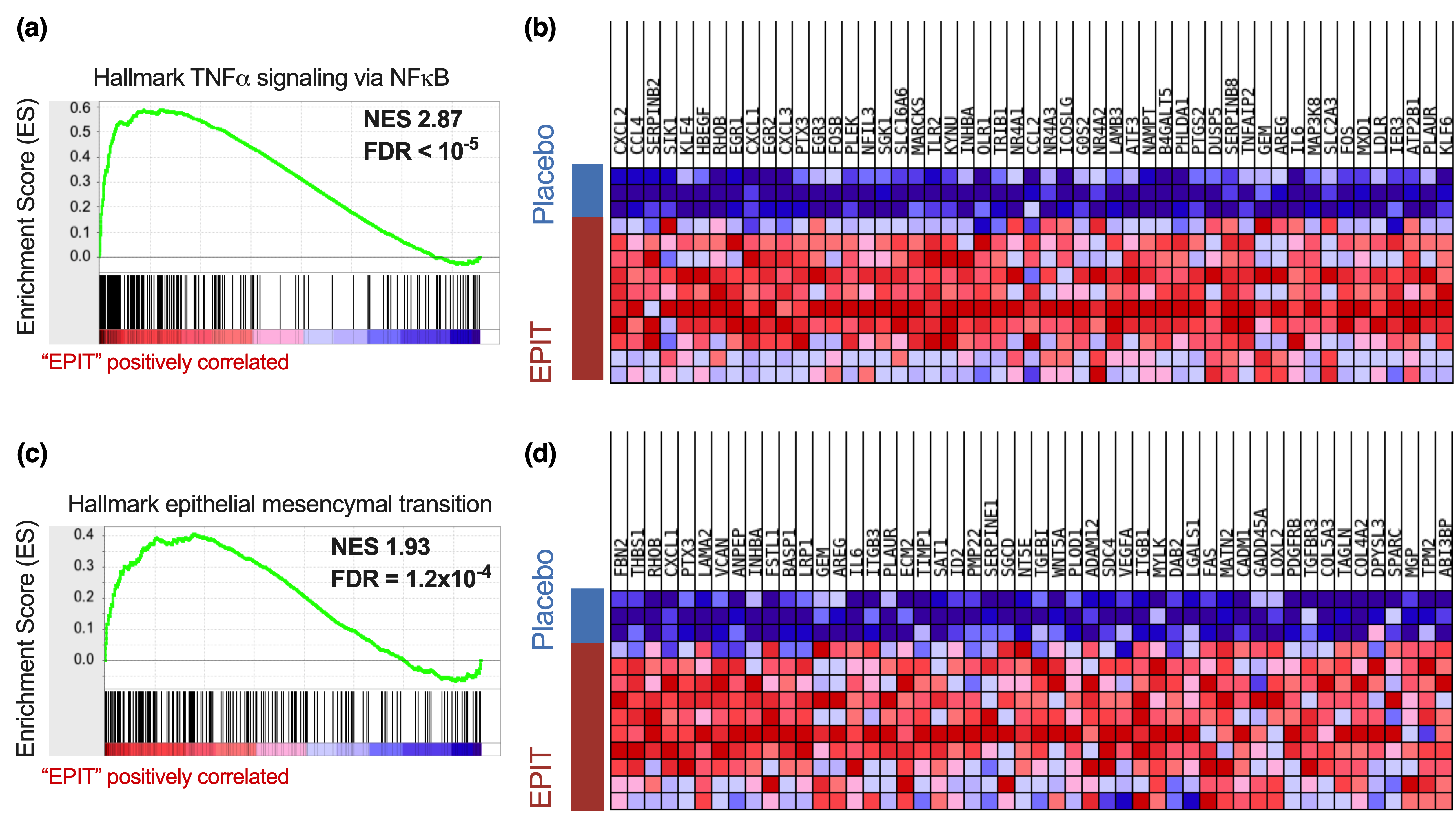
**

**Supplementary Figure 2:** Gene set enrichment analysis (GSEA) was performed with the canonical pathway gene sets in GSEA Molecular Signatures Database using differentially expressed genes of CD4+ cells from patients on milk EPIT (n=10) versus placebo therapy (n=3). **(a)** GSEA Enrichment plot (score curves) **(b)** the heatmap of top 50 contributing enriched genes in the TNFα signaling via NFκB signaling pathway. **(c)** GSEA Enrichment plot (score curves) **(d)** the heatmap of top 50 contributing enriched genes in the Epithelial Mesenchymal transition pathway.

**Supplementary Table 4:** **Differentially expressed genes, EPIT therapy responders versus nonresponders.** Using the Benjamini-Hochberg method with an FDR cutoff of 0.05, we observe a total of 42 DEGs, with 33 upregulated and 9 downregulated transcripts in CD4+ samples collected from EoE patients with favorable (responders, n = 5, post-EPIT biopsy < 15 eos/hpf) vs unfavorable (nonresponders, n = 6, post-EPIT biopsy ≥ 15 eos/hpf) clinical response to EPIT.

| **Gene Symbol** | **Log2(Fold Change)** | **FDR** |
| --- | --- | --- |
| CXCL10 | 2.1496 | 0.023 |
| FCGR3B | 1.8649 | 0.036 |
| OVOS2 | 1.7539 | < 10e^-4^ |
| CCL3L3 | 1.5056 | < 10e^-4^ |
| FEZ1 | 1.4216 | 0.042 |
| NKG7 | 1.2168 | 0.041 |
| CCL2 | 1.1478 | < 10e^-4^ |
| CCL3 | 1.1431 | 0.035 |
| STEAP4 | 1.1371 | 0.035 |
| SIGLEC5 | 1.1211 | 0.036 |
| APOBEC3A | 1.1053 | 0.018 |
| PRSS23 | 1.0951 | 0.036 |
| TNFAIP6 | 1.0934 | 0.036 |
| FCRL6 | 1.091 | 0.042 |
| C19orf59 | 1.0834 | 0.036 |
| FPR1 | 1.0605 | 0.041 |
| HLA-DQB1 | 1.0348 | 0.036 |
| CCL4L1 | 1.031 | 0.042 |
| CCL5 | 1.0211 | 0.045 |
| SIK1 | 1.0004 | 0.014 |
| PTGS2 | 0.9948 | 0.042 |
| HLA-DRB5 | 0.9915 | 0.042 |
| IER3 | 0.9838 | 0.045 |
| EMP1 | 0.9801 | 0.041 |
| LOC101927861 | 0.9669 | 0.023 |
| GJB2 | 0.9625 | 0.045 |
| ZNF319 | 0.9053 | 0.042 |
| GTF2H2B | 0.8446 | 0.035 |
| LOC441081 | 0.8441 | 0.036 |
| LOC100996385 | 0.834 | 0.035 |
| LONRF2 | 0.8242 | 0.041 |
| NAPRT1 | 0.8197 | 0.045 |
| OVOS | -0.8071 | 0.036 |
| ZNF90 | -0.882 | 0.018 |
| RNF17 | -0.9013 | 0.045 |
| CDO1 | -1.0847 | < 10e^-4^ |
| FST | -1.1767 | 0.018 |
| MYT1L | -1.611 | < 10e^-4^ |

**
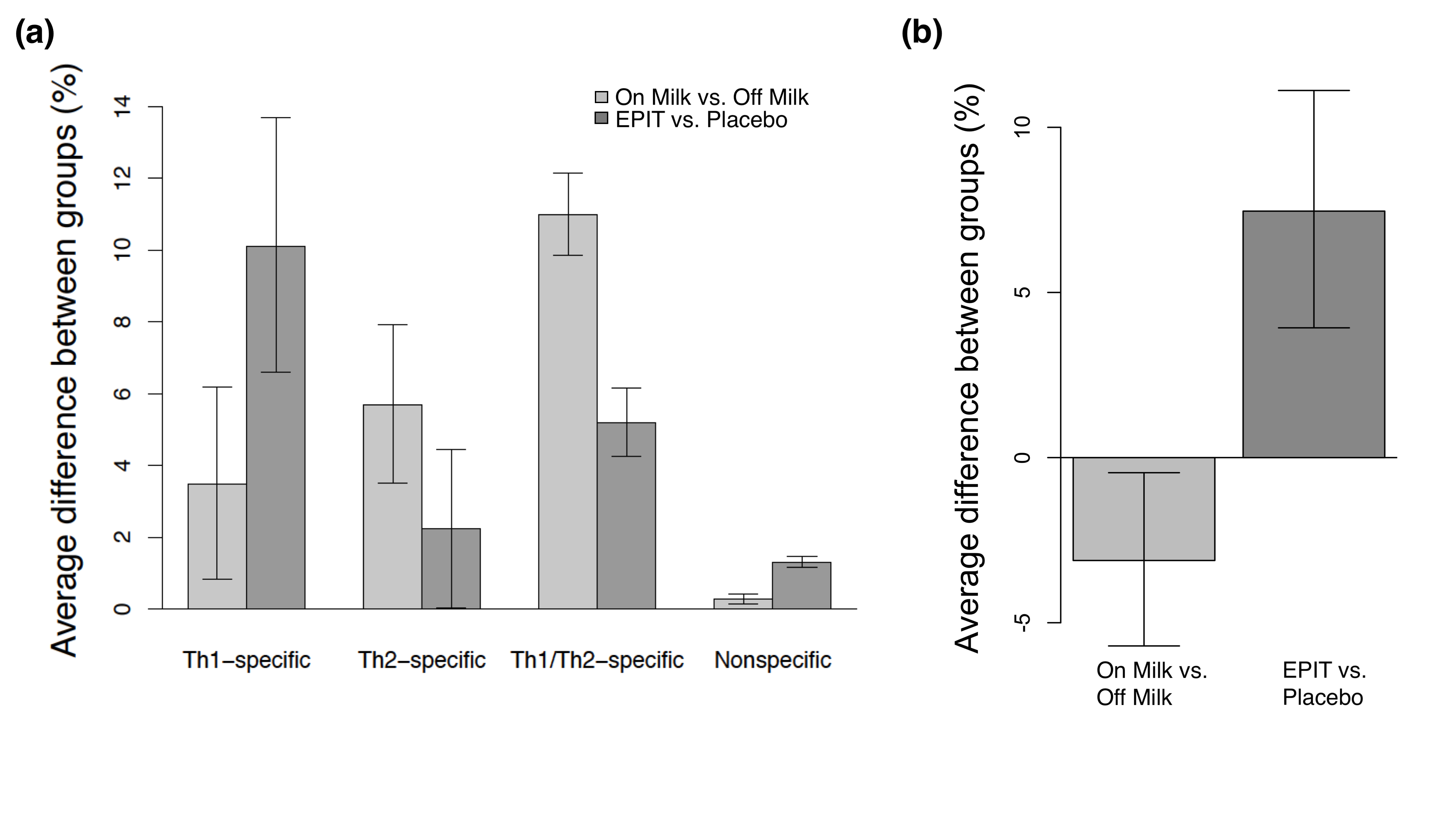
**

**Supplementary Figure 3: Deconvolution analysis of T-cell subset gene signatures suggests EPIT alters balance of T cell subsets.** Published datasets were analyzed to derive unique CD4+ T cell subset signatures for T_helper_ (GSE3982) and T_regulatory_ (GSE107011) cell subsets.^34–36^ For each GEO data set, ANOVA was used to identify genes differentially expressed between all T cell subtypes and Limma was used to identify unique differentially expressed genes for Th1, Th2 and Treg cell types. We calculated average percent change of all genes in our samples across the specific comparisons (On vs off milk and EPIT vs placebo comparisons) for the sets of Th1, Th2 and Treg genes identified (Average Change %). **(a)** Average change in expression of peripheral CD4+ RNA-seq genes following EPIT (blue) has a higher percent average expression of Th1 signature genes, whereas before therapy the percent average expression of Th2-type genes is higher. **(b)** Peripheral CD4+ from patients post-EPIT have a higher percent average expression of genes specific for regulatory T cells.


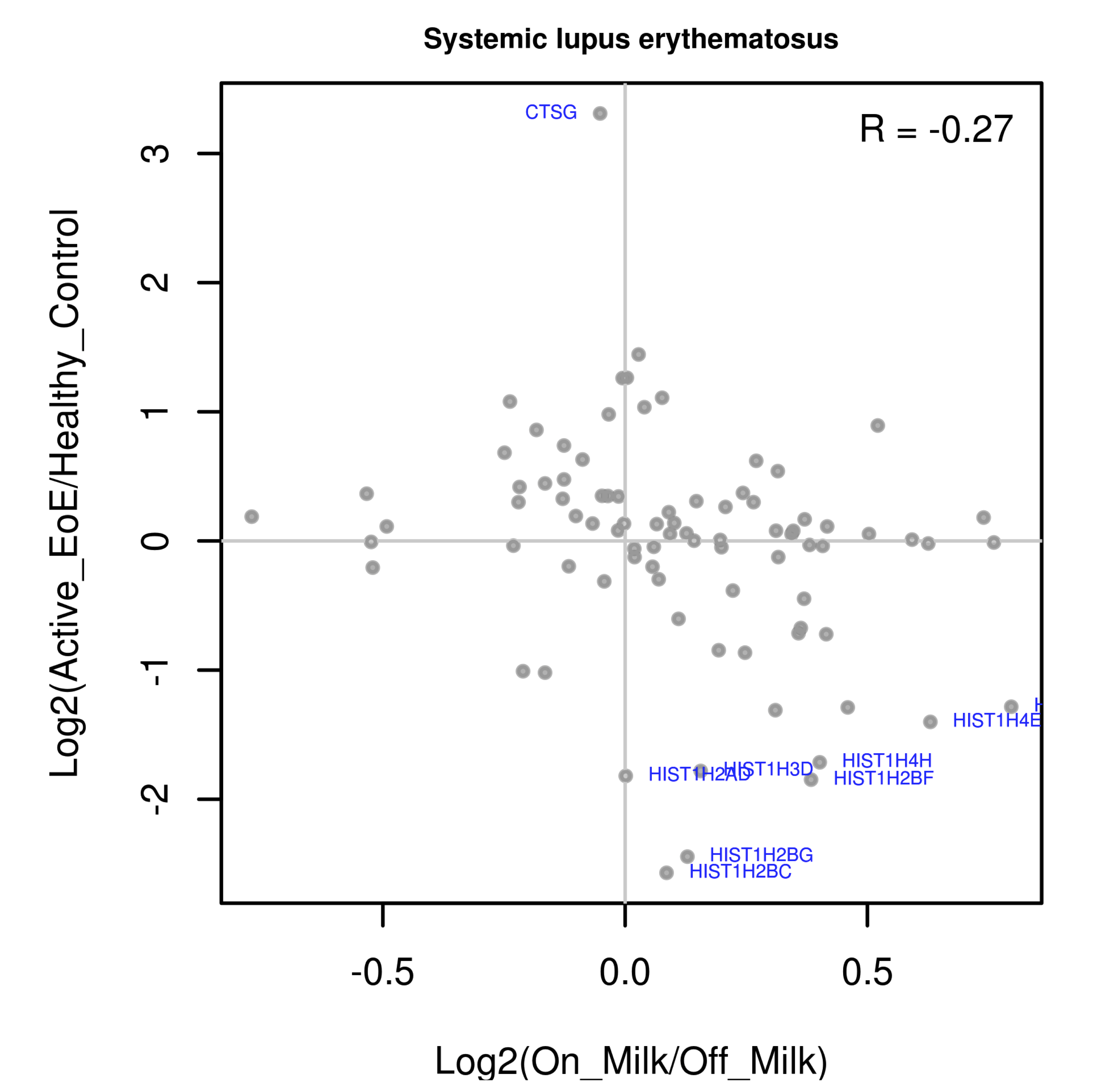
**(a)**

**
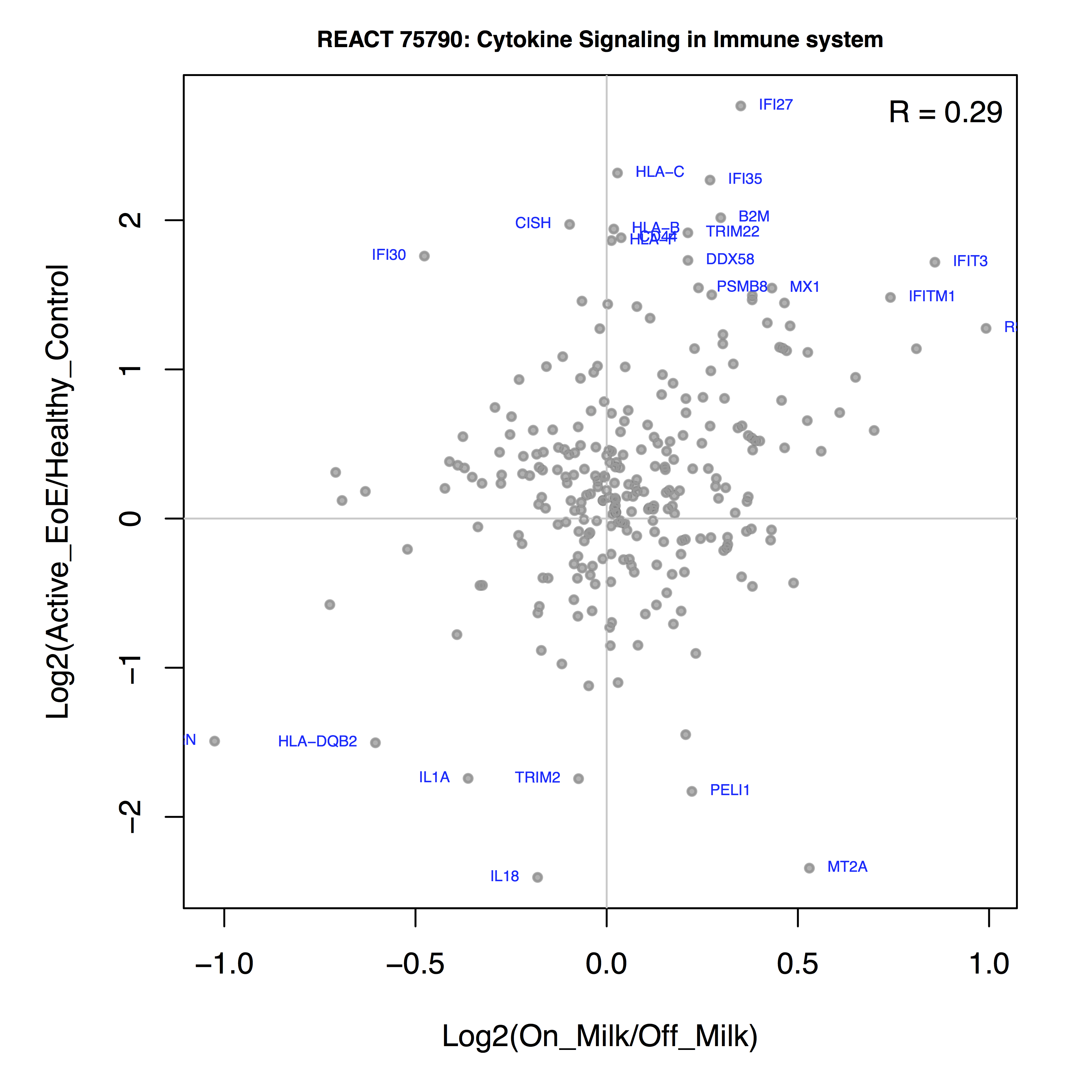
**

**(b)**

**Supplementary Figure 4: Representation of gene expression patterns in biopsy tissue and circulating CD4+ cells in EoE patients during active disease.** Fold change graphs of analysis results for the top 250 common highly expressed transcripts with *P* ≤ 0.01 in the EoE patient peripheral CD4+ samples collected before EPIT, (while on and off of milk containing diet) and the EoE biopsy tissue RNA-seq dataset from Sherrill *et al.*^37^ Results for **(a)** KEGG Systemic lupus erythematosus and **(b)** REACTOME Cytokine signaling in immune system are shown.

**Supplementary Table 5:** **REACTOME pathway analysis results, EPIT responder vs. nonresponder groups.** Reactome pathway analysis was performed using differentially expressed genes from the EPIT nonresponder vs responder comparison meeting FDR < 0.1 cutoff. REACTOME pathways with FDR < 0.1 are reported.^30^

| **Pathway Name** | **Number found** | **Total in Pathway** | **Ratio** | ***P*-value** | **FDR** |
| --- | --- | --- | --- | --- | --- |
| Translocation of ZAP-70 to Immunological synapse | 16 | 42 | 0.003 | 1.11E-16 | 2.44E-15 |
| Phosphorylation of CD3 and TCR zeta chains | 16 | 45 | 0.003 | 1.11E-16 | 2.44E-15 |
| Interleukin-10 signaling | 16 | 86 | 0.006 | 1.11E-16 | 2.44E-15 |
| PD-1 signaling | 16 | 45 | 0.003 | 1.11E-16 | 2.44E-15 |
| Generation of second messenger molecules | 16 | 59 | 0.004 | 1.11E-16 | 2.44E-15 |
| Interferon gamma signaling | 24 | 252 | 0.017 | 1.11E-16 | 2.44E-15 |
| Costimulation by the CD28 family | 16 | 97 | 0.007 | 1.11E-16 | 2.44E-15 |
| Cytokine Signaling in Immune system | 42 | 1,312 | 0.089 | 1.11E-16 | 2.44E-15 |
| Interferon Signaling | 24 | 396 | 0.027 | 6.66E-16 | 1.27E-14 |
| Downstream TCR signaling | 16 | 124 | 0.008 | 8.88E-16 | 1.51E-14 |
| TCR signaling | 16 | 147 | 0.01 | 1.19E-14 | 1.85E-13 |
| MHC class II antigen presentation | 16 | 148 | 0.01 | 1.32E-14 | 1.85E-13 |
| Immune System | 53 | 2,869 | 0.196 | 8.29E-13 | 1.08E-11 |
| Signaling by Interleukins | 18 | 639 | 0.044 | 5.75E-07 | 6.90E-06 |
| Chemokine receptors bind chemokines | 6 | 57 | 0.004 | 3.77E-06 | 4.14E-05 |
| Adaptive Immune System | 19 | 999 | 0.068 | 7.24E-05 | 7.96E-04 |
| Peptide ligand-binding receptors | 8 | 211 | 0.014 | 1.38E-04 | 1.38E-03 |
| Neutrophil degranulation | 12 | 480 | 0.033 | 1.59E-04 | 1.43E-03 |
| Interleukin-33 signaling | 2 | 4 | 0 | 3.91E-04 | 3.52E-03 |
| Formyl peptide receptors bind formyl peptides and many other ligands | 2 | 11 | 0.001 | 2.86E-03 | 2.29E-02 |
| Interleukin-4 and Interleukin-13 signaling | 6 | 211 | 0.014 | 4.05E-03 | 3.24E-02 |

**Supplementary Table 6: Overrepresentation analysis comparison of biopsy and circulating CD4+ gene expression in active EoE.** We examined overlap of differentially expressed genes from EoE patient peripheral CD4+ samples collected before EPIT, (while on and off of milk containing diet) and the EoE biopsy tissue RNA-seq dataset from Sherrill *et al.*^37^ The top 250 genes from the two comparisons with *P* ≤ 0.01 and highest fold change in common genes were analyzed, and results from overrepresentation analysis pathways with *P* ≤ 0.05 are shown.

| **Collection** | **Name** | **Within (*N*)** | **Total (*N*)** | **Within (%)** | **Odds Ratio** | ***P-*Value** | **FDR** | **Direction of Change** |
| --- | --- | --- | --- | --- | --- | --- | --- | --- |
| KEGG_pathway | [Cytokine-cytokine receptor interaction](http://www.genome.jp/dbget-bin/www_bget?pathway+hsa04060) | 8 | 127 | 3.2 | 3.69 | 2.3e-03 | 7.3e-01 | Down; On vs Off Milk |
| KEGG_pathway | [Cytokine-cytokine receptor interaction](http://www.genome.jp/dbget-bin/www_bget?pathway+hsa04060) | 11 | 188 | 4.4 | 4.28 | 1.1e-04 | 3.1e-02 | Up; EoE vs Control |
| GO_BP | [GO:0006954_inflammatory response](http://amigo.geneontology.org/amigo/term/GO:0006954) | 20 | 257 | 8.0 | 4.83 | 1.0e-07 | 2.5e-03 | Down; On vs Off Milk |
| GO_BP | [GO:0006954_inflammatory response](http://amigo.geneontology.org/amigo/term/GO:0006954) | 21 | 345 | 8.4 | 4.62 | 0.0e+00 | 9.8e-05 | Up; EoE vs Control |
| GO_BP | [GO:0030198_extracellular matrix organization](http://amigo.geneontology.org/amigo/term/GO:0030198) | 11 | 177 | 4.4 | 3.67 | 4.2e-04 | 3.8e-01 | Down; On vs Off Milk |
| GO_BP | [GO:0030198_extracellular matrix organization](http://amigo.geneontology.org/amigo/term/GO:0030198) | 11 | 284 | 4.4 | 2.76 | 3.5e-03 | 2.4e-01 | Up; EoE vs Control |
| GO_BP | [GO:0034340_response to type I interferon](http://amigo.geneontology.org/amigo/term/GO:0034340) | 9 | 54 | 3.6 | 11.08 | 5.0e-07 | 1.2e-03 | Up; On vs Off Milk |
| GO_BP | [GO:0034340_response to type I interferon](http://amigo.geneontology.org/amigo/term/GO:0034340) | 15 | 56 | 6.0 | 25.86 | 0.0e+00 | 0.0e+00 | Up; EoE vs Control |
| GO_BP | [GO:1901568_fatty acid derivative metabolic process](http://amigo.geneontology.org/amigo/term/GO:1901568) | 6 | 75 | 2.4 | 5.91 | 8.5e-04 | 1.5e-01 | Up; EoE vs Control |
| GO_BP | [GO:1901568_fatty acid derivative metabolic process](http://amigo.geneontology.org/amigo/term/GO:1901568) | 6 | 75 | 2.4 | 5.91 | 8.5e-04 | 6.7e-01 | Down; EoE vs Control |
| KEGG_pathway | [Systemic lupus erythematosus](http://www.genome.jp/dbget-bin/www_bget?pathway+hsa05322) | 11 | 66 | 4.4 | 11.17 | 0.0e+00 | 9.2e-05 | Up; On vs Off Milk |
| KEGG_pathway | [Systemic lupus erythematosus](http://www.genome.jp/dbget-bin/www_bget?pathway+hsa05322) | 6 | 88 | 2.4 | 4.97 | 1.9e-03 | 7.6e-01 | Down; EoE vs Control |
